# Why is the world not yet ready to use alternative fuel vehicles?

**DOI:** 10.1016/j.heliyon.2021.e07527

**Published:** 2021-07-14

**Authors:** Meisam Ahmadi Ghadikolaei, Pak Kin Wong, Chun Shun Cheung, Jing Zhao, Zhi Ning, Ka-Fu Yung, Hang Cheong Wong, Nirmal Kumar Gali

**Affiliations:** a Department of Electromechanical Engineering, University of Macau, Taipa, Macau; b Department of Mechanical Engineering, The Hong Kong Polytechnic University, Hung Hom, Kowloon, Hong Kong; c Division of Environment and Sustainability, The Hong Kong University of Science and Technology, Clear Water Bay, Hong Kong; d Department of Applied Biology and Chemical Technology, The Hong Kong Polytechnic University, Hung Hom, Kowloon, Hong Kong

**Keywords:** Alternative fuel and vehicle policy, Alternative fuel vehicles, Clean fuels, Consumer purchase intention, Environmental concern, Gasoline and diesel vehicles

## Abstract

Despite the improvement in technologies for the production of alternative fuels (AFs), and the needs for using more AFs for motor vehicles for the reductions in air pollution and greenhouse gases, the number of alternative fuel vehicles (AFVs) in the global transportation sector has not been increasing significantly (there are even small drops for adapting some AFs through the projections) in recent years and even in the near future with projections to 2050. And gasoline and diesel fuels will remain as the main energy sources for motor vehicles. After reviewing the latest advantages and disadvantages of AFVs, including flexible-fuel, gas, electric, hybrid electric, and fuel cell electric vehicles, it is found that the higher price of AFVs, compared to that of gasoline and diesel vehicles, might be one of the main barriers for their wider adoption. But on the other hand, there is the “chicken and egg” problem. Because people mostly do not select AFVs due to their higher price and sometimes their less infrastructure availability compared to those of gasoline and diesel vehicles, however, governments and AFVs manufacturers claim that the insignificant demand volume and less interest by people to purchase them, is one of the main reasons for a higher price and less infrastructure availability of AFVs. So, what should we do for adopting AFVs? This review shows that there are two very important and fundamental points that might cause a rise in the demand and usage of AFVs, rather than waiting for the reduction in AFVs prices. Those points are car salespeople's and people's knowledge about AFVs and the environmental issues, and their encouragement to accept and use AFVs. Although the AFVs are available on the market for many years, many people around the world have no/less/old/wrong knowledge about the current AFVs. Thus, most of these people reject these vehicles for usage, even when their important parameters such as purchase price, operating cost, driving range, and fuel availability be the same (or close) as those of gasoline or diesel vehicles. Detailed information, examples, and recommendations to the increases in people's knowledge and encouragement are presented in this review.

## Highlights

• Latest advantages and drawbacks of alternative fuel vehicles (AFVs) are presented.
• AFVs include flexible-fuel, gas, electric, hybrid, and fuel cell electric vehicles.
• People's knowledge and encouragement on AFVs are important for wider AFVs adoption.
• Several technical recommendations for increase in AFVs adoption rate are presented.
• This review is useful for the researchers, governments, and policymakers on AFVs.

## 1 Introduction

**Figure 1:**
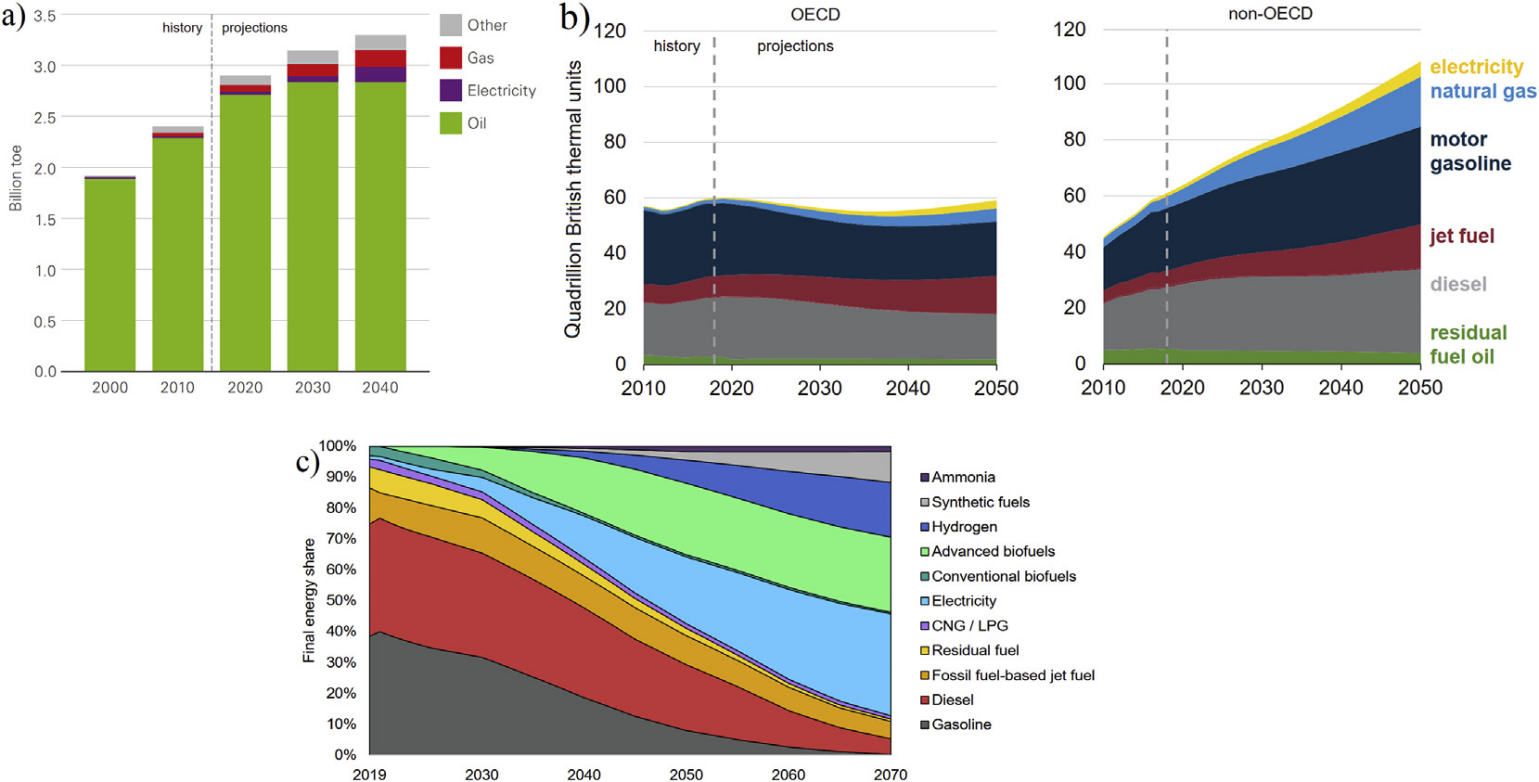
Energy consumption in global transportation projected by a) BP Energy Outlook up to 2040 according to Evolving Transition Scenario (the figure is reprinted from Ref. [4] with permission from BP), “Other” includes biofuels, coal, and hydrogen, and “toe” stands for tonne of oil equivalent, b) International Energy Outlook up to 2050 according to Reference Case Scenario (the figure is reprinted with permission from EIA “Source: U.S. Energy Information Administration (Sep 2019) [1]”), OECD stands for countries in part of the Organization of Economic Cooperation and Development, and c) International Energy Agency reported in 2020 for 2019–2070 according to Sustainable Development Scenario (the figure is reprinted with permission from IEA “Source: IEA (2020) Energy technology perspectives 2020. All rights reserved [3]”).

**Figure 2:**
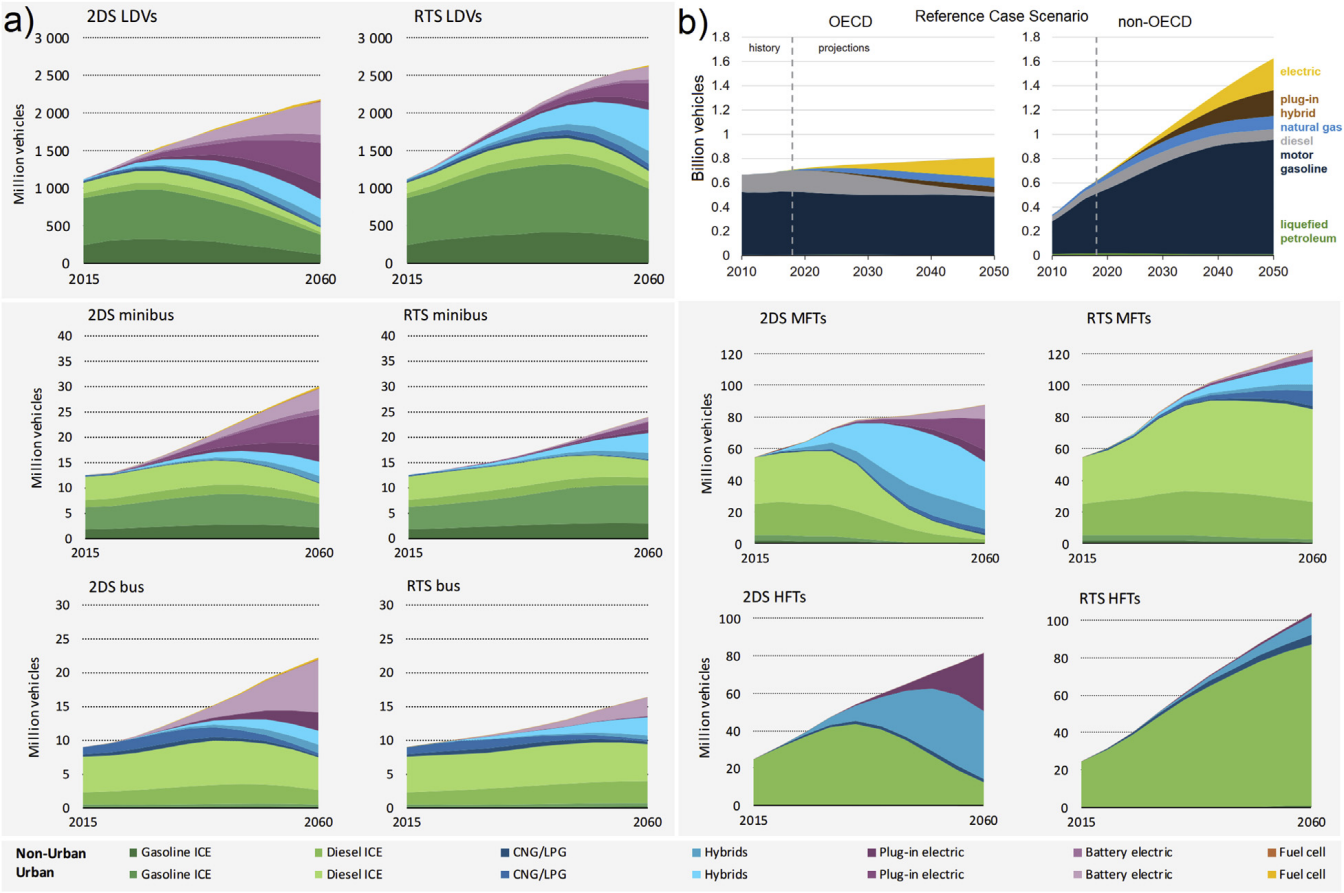
Global vehicle stock by types, fuels, and projection scenarios for a) International Energy Agency reports in 2017 according to 2 °C Scenario (2DS) and Reference Technology Scenario (RTS) for light-duty vehicles (LDVs), minibus, bus, and medium and heavy freight trucks (MFTs and HFTs) (the figures are reprinted with permission from IEA “Sources: IEA (2017) Mobility model March 2017 version, database and simulation model. All rights reserved [7] and IEA (2017) Energy technology perspectives 2017. All rights reserved [8]”) and b) International Energy Outlook in 2020 according to Reference Case Scenario for light-duty vehicles (the figure is reprinted with permission from EIA “Source: U.S. Energy Information Administration (Sep 2019) [1]”), OECD stands for countries in part of the Organization of Economic Cooperation and Development.

**Figure 3:**
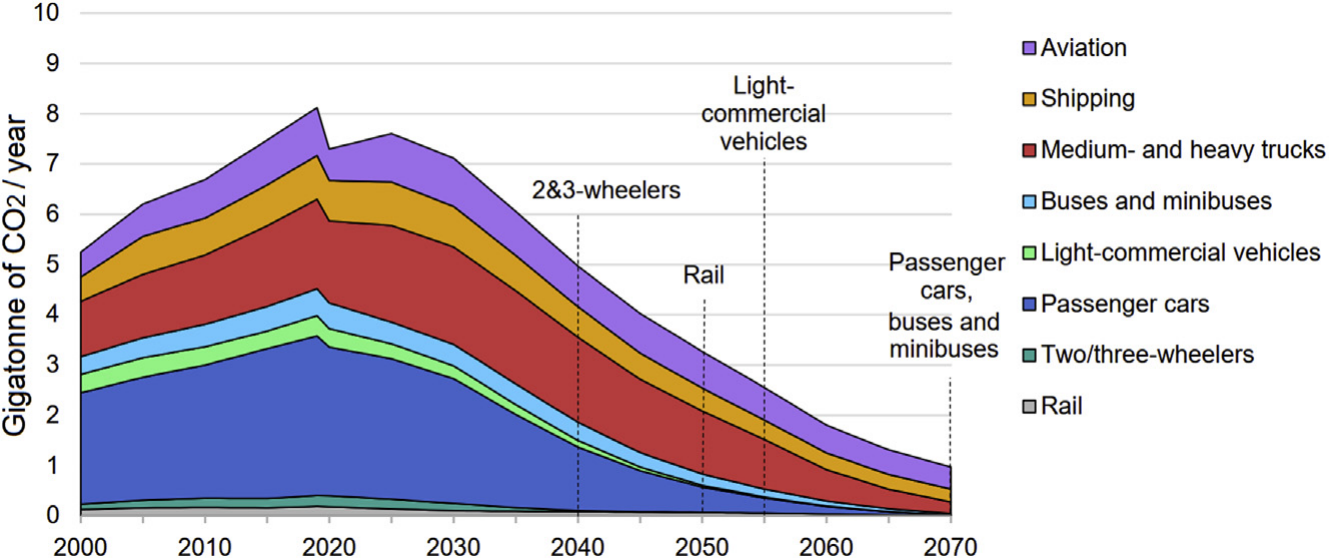
Global CO_2_ emissions in transport by mode according to Sustainable Development Scenario reported in 2020 for 2000–2070 (the figure is reprinted with permission from IEA “Source: IEA (2020) Energy technology perspectives 2020. All rights reserved [3]”). Note: dotted lines represent the year in which various transport modes have largely stopped consuming fossil fuels, resulting in no longer contribute to direct CO_2_ emissions from the combustion of fossil fuels.

The history of using modern vehicles goes back to over 130 years, while fossil fuels were used as the main energy sources. Since then, from time to time, the inventors and researchers tried to replace fossil fuels with some limited AFs, ethanol as an example, for various reasons. But they did not achieve very good success due to less developed technologies available and no existence of big air pollution and greenhouse gas (GHG) emissions issues (compared with today) as motivators in those periods, therefore, fossil fuels remained the main sources. While in recent decades, the world is suffering from air pollution and global warming due to the burning of fossil fuels, among other reasons. Also, the technologies available in recent years to produce AFVs are more developed than before (130 years ago) which causes the generation of several types of AFVs, such as flexible-fuel, gas, electric, hybrid electric, fuel cell vehicles, etc. However, the data in the global transportation sector (more than 70% by vehicles [1, 2]) shows that the contribution (both energy consumption and number) of AFVs available nowadays and even for the near future (projected up to 2050 as the net-zero emissions target year in terms of greenhouse gases in many countries [3]) is not as high as the contribution required to reduce air pollution and dependency to the usage of gasoline and diesel vehicles. And, despite the increase in using AFs for vehicles (particularly electricity and natural gas), gasoline and diesel are/will be the main sources of energy for the transportation sector in recent years/the near future with projections to 2050 [1, 2, 4, 5, 6]. It is projected that the contribution of oil, as a main transportation energy source, in the global transportation fleets, will drop from 94% at present [1, 4] to only about 85% by 2040 according to Evolving Transition Scenario^1^ [4], about 82% by 2050 according to Reference Case Scenario^2^ [1], or about 40% by 2050 according to Sustainable Development Scenario^3^ [3] as shown in Figure 1. In the aspect of the number of AFVs in the global transportation fleets, it is reported that the current number of global vehicles (about 1 billion) will reach 2–2.5 billion by 2050, while AFVs will be counted as less than half of the overall quantity in 2050 and oil will continue to be used in the future according to Reference Case, Reference Technology^4^, and 2 °C^5^ Scenarios, as shown in Figure 2. Regarding GHG emissions form the global transportation fleets, the latest report in 2020 [3] illustrates that global CO_2_ gas (as the main contributor of GHG emissions) will be reduced in the future, say about 60% reduction in 2050 compared to 2019 by applying different policies and actions, but it will not be zero by 2050 and even by 2070, while alternative fuels will have about 70% contribution in the reduction of CO_2_ emissions in 2070 as shown in Figure 3. Therefore, reducing CO_2_ emissions in the transportation sector over the coming half-century is a formidable task [3].

On the one hand, the serious attempt to use AFs is started since several years ago (e.g., the Clean Air Act in 1970 as a key piece of domestic environmental legislation, and the Energy Policy Act of 1992 (EPAct 1992) for establishing voluntary and mandatory programmatic activities to increase using AFs [9]), but only 6% of the global transportation energy source has been shifted to AFs to date. On the other hand, the huge number of conducted studies reported in the literature using AFs for vehicles shows that the attention to the use of AFs by the researchers is rising annually. However, the rise in implementation of AFs for vehicles is not as fast as this annual rise in the attention of using AFs, because only 6% of the current global transportation fleets use AFs [1, 4]. The insufficient implementation to the use of AFs for vehicles can be investigated through different aspects, including fuels (price, availability, production, etc.), vehicle performance and emissions, users, governments, human health, and the environment. In addition, despite the increase in technology development for the production of AFs (causing a decrease in the final product price and an increase in product availability for the users) year by year, the number of AFVs existed in the transportation sector is almost less than the projected number for every year and decade as reported by the reputed institutions and publications in the global energy fields, like International Energy Outlook [1] and Annual Energy Outlook [6] published by Energy Information Administration (EIA), Outlook for Energy published by ExxonMobil [2], Energy Technology Perspectives published by International Energy Agency (IEA) [3], BP Energy Outlook published by British Petroleum (BP) [4], World Oil Outlook published by Organization of the Petroleum Exporting Countries (OPEC) [5], etc., which have projections to 2050. For instance, the old version of Outlook for Energy (2015) reported that the energy demand for the global transportation sector will be sourced by oil with the contribution of 113 and 124 quadrillion British thermal units (Btu), and AFs (including biodiesel, gas, and others) with the contribution of 10 and 16 quadrillion Btu in 2025 and 2040, respectively [10]. While it reported in 2017 that oil will be used as 116 and 123 quadrillion Btu, and the aforementioned AFs will be used as 9 and 15 quadrillion Btu in 2025 and 2040, respectively [11]. However, its latest version (2019) reported that oil will be used as 116 and 121 quadrillion Btu, while those AFs will be used as 9 and 19 quadrillion Btu in 2025 and 2040, respectively [2].

In another example, as a small scale for one leading country in the AFVs application, the 2018 Annual Energy Outlook reported that the use of minor petroleum and AFs, including electricity, compressed and liquefied natural gas, residual fuel oil, pipeline fuel natural gas, other petroleum, propane, and hydrogen in the US transportation sector will be about 1.7 quadrillion Btu in 2019 and about 2.8 quadrillion Btu in 2050 [12]. However, its latest version (2020) reported that the contribution of those fuels became only about 1.5 quadrillion Btu in 2019 and it will be about 2.3 quadrillion Btu in 2050 [6]. We used the above examples to illustrate that despite improvement in technologies year by year for AFs production and more needs for using AFs for the reductions in air pollution and GHG emissions [3, 13, 14, 15, 16], the number of AFVs in the transportation sector is not rising significantly (even there is a small drop) for every year and in the near future. Therefore, there should be some important missing parameters that are more effective than the technology development (as the main factor influencing AFVs cost) for AFs production (fuel price, availability and production, and vehicle performance) on the contribution of using AFVs. This review tries to find them and answer the following question with some recommendations for a solution: why is the contribution of AFVs still very low in the global transportation sector compared with gasoline and diesel vehicles, despite existing huge air pollution issue, more interest to use AFs by governments and researchers, and more availability of technologies for the production of AFs?

In addition, it might be right that the technologies for the production of the AFVs will be more developed in the future, resulting in the reduction in their price and more availability and safety; however, on the other hand, the technologies on the conventional gasoline and diesel vehicles will also be more improved, resulting in changes in their fuel economy, performance and emissions (e.g., using more efficient catalytic converters and using CO_2_ capture system for vehicles), and also their more global availability (especially in less developed countries which have significant population) in the future, resulting in offsetting environmental benefits of AFVs [17, 18]. Also, some of the AFs have a small or indirect dependency on the price of crude oil; however, the price of gasoline or diesel is strictly and directly dependent on the price of crude oil [19]. Thus, projection of gasoline and diesel price for the future will be a difficult task due to huge changes in crude oil price annually (e.g., global oil price dropped from USD 80/barrel to USD 50/barrel from early 2018 to the end of 2018 [5], and even it was less than USD 50/barrel in several months in 2020), therefore, gasoline and diesel fuels price may become less than today's price or even less than the price of some AFs in the future, resulting in remaining gasoline or diesel vehicles attractive for most buyers [20]. For example, the global price of gasoline and diesel, as a large scale, was firstly increased by about 20% from 2010 to 2012, then remained almost unchanged until 2014, and finally dropped by about 30% in 2016 which is almost the same as crude oil price’ trend, with different magnitudes, during the same period (that period had a huge price fluctuation in recent years) [5, 21, 22]. Similarly, the US gasoline and diesel fuel price, as a small scale, had the same trend, with about 30% drops, almost remaining unchanged, and about 40% increases during the same period [23]. On the other hand, despite the significant influence of vehicle and fuel price on consumer decision-making for selecting vehicles, the non-price attributes can also play an important and significant role in consumer decision-making [24, 25]. Thus, among these uncertainties, what guarantee is available that shows people will select AFVs in the future, rather than selecting gasoline and diesel vehicles. This review also aims to find possible and permanent solutions for that through answering the following question: how can we achieve a greater AFVs number than the projection number in the future (say in 2050 or later) for sure, instead of probability?

## 2 Review method

This review has been completed after several steps as follows. In the first step, we monitored and tracked the past, current, and future trends of the AFVs adoption in the global transportation fleets through the international energy reports such as International Energy Outlook and Annual Energy Outlook published by EIA, Outlook for Energy published by ExxonMobil, Energy Technology Perspectives published by IEA, BP Energy Outlook published by BP, World Oil Outlook published by OPEC, etc., from 2015 onwards according to different projection scenarios, including Evolving Transition, Reference Case, Sustainable Development, Reference Technology, and 2 °C Scenarios. Then, we found that the current AFVs adoption rate will not meet the goal of the projected year of 2050 as the net-zero emissions target year in terms of greenhouse gases and decarbonizing road transport in many countries, under any scenario (as mentioned in the Introduction section). In the second step, the current types of AFVs available in the global transportation fleets, including flexible-fuel, gas, electric, hybrid electric, and fuel cell electric vehicles were identified along with their latest advantages and disadvantages over gasoline and diesel vehicles (Table 1). Since the advantages and disadvantages of AFVs are changing over time due to the improvement in their production technologies, we gathered their most recent advantages and disadvantages from 2016 to 2021 (mostly 2020).

**Table 1:** Major benefits and drawbacks of AFVs compared to gasoline and diesel vehicles up to today's technologies (2020–2021).

Type of vehicle	Benefits	Drawbacks
**Flexible-fuel vehicles (ethanol)** [26, 31, 37, 41]	• Ethanol is a renewable fuel	• Higher fuel cost than gasoline
	• Reduction in gasoline fuel consumption	• Impact on food cost and availability (for edible feedstocks)
	• No fuel sulfur and aromatics content	• Cold starting problem for high ethanol concentration in cold climates
	• Resistance to knocking and improve combustion due to higher octane rating than gasoline	• Need for modifications of current gasoline engines for using more than 10% of ethanol
	• Reduction in exhaust emissions, especially PM	• Lower fuel economy (distance traveled by a vehicle and the amount of fuel consumed) due to lower energy density (GGe and Btu/gallon) than gasoline, E10 by 3–4%, E15 by 4–5% and E85 vehicles by 15–27% have fewer MPG than pure gasoline vehicle
	• Almost no need for modifications of current gasoline engines up to using 10% of ethanol	• Limited availability of ethanol on a worldwide scale (currently, only some countries have the feedstocks and technologies for ethanol production on large scales)
	• Reduction in GHG emissions (CO_2_) through life-cycle basis due to using biomass feedstocks	
**Flexible-fuel vehicles (biodiesel)** [27, 28, 37, 42, 43, 44]	• Biodiesel is a renewable fuel	• Increase in NOx
	• Reduction in exhaust emissions such as CO, HC, and PM	• Higher fuel cost than diesel
	• Reduction in GHG emissions (CO_2_) through life-cycle basis due to using renewable feedstocks, and reduction in GHG emissions by 15% for B20 and 74% for pure biodiesel compared with diesel fuel	• Concerns about food versus fuel
	• Reduction in diesel fuel consumption	• Impact on food cost and availability (for edible feedstocks biodiesel)
	• Almost no fuel sulfur and aromatics content	• Deforestation for oil crops plantation
	• Almost no need for modifications of current diesel engines up to using 20% of biodiesel	• Need for modifications of current diesel engines for using more than 20% of biodiesel
	• Almost similar engine power when using B20 or lower biodiesel percentage compared with diesel fuel	• Causing contamination of fuel storage tanks like microbial growth and sludge formation
	• Less damage impact than diesel fuel if biodiesel spilled or released to the environment	• Increase the chance of fuel filter clogging and also injector fouling
	• Safer to handle, store and transport than diesel	• Reduction in engine power and fuel economy when using more than 20% of biodiesel due to its lower energy density (Btu/gallon) than diesel (lower fuel economy and power of 10% for B100, and 2% for B20)
	• Improving fuel lubricity (increase engine's moving parts lifetime) and raises the cetane number (better combustion quality) of the blend	• Quality of biodiesel varies widely due to using various feedstocks types
		• Using high biodiesel concentration (more than 20%) may damage the rubber and plastics parts in the fueling system or carbon build-up in the engine
		• Use of blends above B5 has not yet been widely approved by many automakers
**Natural gas vehicles (CNG and LNG)** [22, 26, 35, 37, 45, 46]	• Offsetting upfront costs for converting traditional vehicles to the natural gas vehicles due to lower fuel, operating and maintenance costs over the natural gas vehicles' lifespan	• Purchase prices can be slightly higher than traditional gasoline or diesel vehicles, while 50–75% of this higher price is due to the fueling system for natural gas. But since natural gas is typically cheaper than gasoline fuel, thus, the return on investment can be quick
	• Reduction in GHG emissions (CO_2_) for both direct basis (from vehicle) and life-cycle basis compared to those of traditional gasoline or diesel vehicles; for example, light-duty natural gas vehicles reduce by 6–11% in GHG compared to gasoline throughout the fuel life-cycle	• Need for fuel (gas or liquid) storage tanks inspection by qualified service facility at regular intervals (for example, at least every three years or every 36,000 miles for CNG) for safety issue
	• Reduction in tailpipe emissions	• Higher risk for ignition of leaking fuel (which is in gas form) during traffic accidents
	• Extending the useful life for the engine's lubricating oil due to burning less carbon available in natural gas	• CNG is sensitive during refueling to some conditions such as fueling rate, pressure rating, ambient temperature, etc.; for instance, hot ambient temperature with using fast-fill method can cause filling only 75% of design potential capacity of fuel tank (if a proper filling pressure adjustment system is nor used in the fuel station)
	• Comparable horsepower, acceleration, and cruise speed of natural gas vehicles compared to diesel or gasoline vehicles	• Lower driving range (on a single fuel tank) of natural gas vehicles compared to diesel or gasoline vehicles, because of the lower energy density of natural gas (GGe and Btu/gallon)
	• Natural gas is cheaper than gasoline or diesel fuel	• Although natural gas has almost the highest number of fueling stations among the AFs, it has lower fueling infrastructure compared to oil (gasoline or diesel) stations
	• Natural gas vehicles can save about up to 50% per gallon at the pump price compared to gasoline or diesel vehicles	• CNG vehicle has 10–15% loss of engine output power than gasoline vehicle
	• Natural gas causes having cleaner and longer-lasting engines (spark plugs, engine oil, and engine cylinders)	• More fueling time required (minutes to hours) than gasoline or diesel vehicles, if CNG fueling system is in time-fill mode; for fast-fill mode, the fueling time (less than 5 min, as 20 gallons of gasoline) of CNG is almost the same as gasoline or diesel
	• Resistance to knocking and improve combustion due to higher octane rating of natural gas than gasoline	• Natural gas vehicles emit methane (a greenhouse gas which is several times worse than CO_2_ on global warming), after-treatment system on vehicle can reduce methane
**Liquefied petroleum gas vehicles (LPG), also known as propane vehicles** [26, 29, 37]	• Resistance to knocking and improve combustion due to higher octane rating than gasoline	• LPG vehicles can cost several thousand dollars more than comparable traditional gasoline vehicles. But since LPG fuel is typically cheaper than gasoline fuel, thus, the return on investment can be quick
	• Comparable driving range to conventional gasoline vehicle	• Needing more fuel by volume (lower fuel economy) to drive the same distance as gasoline vehicle due to its lower energy density (GGe and Btu/gallon) than gasoline fuel (propane has 27% less energy than gasoline per one gallon). However, lower per-gallon cost of LPG can quickly offset its lower fuel economy
	• Lower maintenance costs of LPG vehicle compared to gasoline vehicle, makes it a choice for high-mileage travels	• Lower fueling infrastructure available, compared to oil (gasoline or diesel) stations
	• Improving in engine life for LPG vehicle, due to higher octane rating, and lower-carbon and lower oil-contamination characteristics compared to those of gasoline fuel	• LPG vehicle in dedicated method (engine run only on LPG fuel) has fewer or sometimes comparable driving range (miles) on a tank of fuel compared to gasoline vehicle
	• Reduction in cold-start issues and even almost no need for using an enriched fuel mixture during cold-weather startups. Because the fuel's mixture in LPG vehicle is completely in gaseous form (propane and air) when entering the engine's cylinder	• Converting a vehicle to use LPG is expensive. But upfront costs (several thousand dollars, e.g., USD 6,000–12,000) for converting traditional vehicles to the LPG vehicles can be offset due to lower fuel, operating, and maintenance costs compared to those of gasoline vehicles over the LPG vehicles' lifespan
	• Reduction in GHG emissions for both direct basis (from vehicle) and life-cycle basis (about 13%) compared to those of traditional gasoline vehicle	• Natural gas production as a resource of LPG creates methane (a greenhouse gas which is several times worse than CO_2_ on global warming)
	• Reduction in tailpipe emissions	
	• Reduction in petroleum use by 99%, if propane be a by-product of natural gas production	
	• LPG vehicle's power, acceleration, and cruising speed are almost similar to those of gasoline vehicles	
	• LPG vehicle in bi-fuel method (engine run on LPG and gasoline fuels) has comparable or higher (if large fuel tank is used) driving range on a tank of fuel compared to gasoline vehicle	
**Battery electric vehicles (BEVs)** [17, 26, 29, 36, 37, 47, 48, 49, 50, 51, 52, 53]	• Possibility of using home, retail, or public charging stations for EVs, rather than the only fueling option (public station) available for gasoline or diesel which is far from home (wasting energy and increase emissions to vehicle fueling from home to the station)	• Purchase prices can be significantly higher (about USD 8,000–16,000 higher, twice or more for some models due to using very expensive battery) than comparable gasoline or diesel vehicles. But their prices are likely to drop as production and demand volumes rise and battery technologies continue to mature
	• Using both renewable and non-renewable sources for electricity production such as oil, coal, natural gas, nuclear, hydropower, wind, solar, biofuels, etc.	• Shorter driving range (per single charge, mostly about 100–200 miles, and 200–300 miles for some vehicles) than comparable conventional gasoline and diesel (per single fuel tank, at least 300 miles)
	• Generation of electricity to recharge the battery from energy harnessed called “regenerative braking” during braking which can rise the range of EVs up to 5% and reduce the heat during braking, resulting in drops in brake wear and maintenance costs	• Charging time for BEVs (mostly 3–12 h, even a "fast charge" to 80% capacity can take about 30 min) is much higher than traditional vehicles' fueling time (about 2–3 min)
	• Reduction in propulsion components; EVs use only an electric traction motor (to provide power to the wheels) and a controller (to control the application of power), while ICEs need several propulsion components such as engine, fueling system, pumps, starter, and intake and exhaust systems	• Lack of sufficient retail charging infrastructure compared to oil (gasoline or diesel) stations
	• Require less maintenance than conventional vehicles	• Time required to offset the higher purchase price of EVs through savings made in ownership costs is long (at least about five-year period relative to an equivalent ICE vehicles)
	• Zero GHG emissions for direct basis (from vehicle) and reduction in GHG emissions on well-to-wheel life cycle basis (from electricity power plants which use fossil or non-fossil fuels) compared to those of ICEs	• Production of EVs needs more CO_2_ formation than that of traditional vehicles, due to batteries production for EVs
	• Zero tailpipe emissions and hence no need for using catalytic converter which has some costs for conventional vehicles	
	• Electricity is much cheaper than gasoline or diesel fuel	
	• Significant batteries' lifetime, today's batteries may last 12–15 years in moderate climates (8–12 years in extreme climates)	
	• Better fuel economy with achieving over 100 MPGe (drive 100 miles consuming only 25–40 kWh; note that according to US EPA, 33.7 kWh of electricity is the equivalent to one gallon of gasoline) compared to traditional ICE vehicles (mostly about 33 MPG)	
	• Operate quieter and smoother and stronger acceleration	
	• BEVs are more suitable than conventional vehicles in using start/stop system	
**Hybrid electric vehicles (HEVs) and plug-in hybrid electric vehicles (PHEVs)** [26, 29, 37, 51, 54]	• ICEs for hybrid vehicles are smaller than traditional ICEs, resulting in lower engine's maintenance cost	• Purchase prices can be significantly higher than traditional gasoline or diesel vehicles (PHEVs are roughly USD 4000–8000 more than a comparable non-plug-in hybrid). But their prices are likely to drop as production and demand volumes rise and battery technologies continue to mature
	• Reduction in vehicle gasoline or diesel fuel consumption (if using ICEs for HEVs and PHEVs (roughly 30–60% lower)), and zero vehicle gasoline or diesel fuel consumption (if using only electricity, not ICEs, for PHEVs)	• Both HEVs and PHEVs have shorter or comparable driving range (per single charge and fueling) than comparable conventional gasoline and diesel (per single fuel tank, at least 300 miles)
	• PHEVs are capable of being powered solely by electricity, while electricity can be produced from several resources such as natural gas, coal, nuclear energy, renewable, etc., resulting in no need for using gasoline or diesel fuel	• Charging time for PHEVs is much higher (mostly 1–4 h, even "fast charge" to 80% capacity may take about 30 min) than traditional vehicles' fueling time (about 2–3 min)
	• Generation of electricity to recharge the battery from energy harnessed called “regenerative braking” during braking which can rise the range of EVs up to 5% and reduce the heat during braking, resulting in drops in brake wear and maintenance costs	
	• Reduction in GHG emissions for both direct basis (from vehicle) and well-to-wheel basis (from electricity power plants which use fossil or non-fossil fuels) compared to those of traditional gasoline or diesel vehicles	
	• Reduction in tailpipe emissions if ICEs work, and zero tailpipe emissions when are in all-electric mode	
	• Electricity for PHEVs is much cheaper than gasoline or diesel fuel	
	• Significant batteries' lifetime, today's batteries may last 12–15 years in moderate climates (8–12 years in extreme climates)	
	• There are available stations as the same fueling infrastructures as gasoline and diesel fuels, because both HEVs and PHEVs can use the same fueling stations as gasoline or diesel vehicles	
	• Better fuel economy with achieving over 100 MPGe (drive 100 miles consuming only 25–40 kWh) compared to traditional ICE vehicles (mostly about 33 MPG)	
	• Operate quieter and smoother	
**Fuel cell electric vehicles (FCEV)** [26, 29, 37, 38, 55, 56, 57]	• Better fuel economy with achieving over 50 MPGe compared to traditional ICE vehicles (mostly about 33 MPG)	• Lower/comparable driving range than/with ICEs; FCEVs can achieve more than 300 miles (mostly 300–500 miles) on a single tank (fueling hydrogen in about 5 min) compared to today's traditional ICE vehicles (at least 300 miles on a single fuel tank)
	• Increase in engine efficiency, fuel cell can achieve 40–70% energy efficiency which is substantially greater than (almost twice) the 30% efficiency of the most efficient ICEs	• Although the first cost of FCEVs has decreased significantly since its concept and prototype time, it is still more expensive than the current conventional ICE and even hybrids vehicles
	• Hydrogen (as a fuel for FCEVs) contains almost three times more energy per unit of weight than current conventional gasoline (one kg of hydrogen is roughly equivalent to one gallon of gasoline)	• Lack of fueling infrastructure for producing, delivering, and dispensing hydrogen (main fuel for FCEVs) to consumers
	• Decrease in smog-forming emissions	• More concern regarding the durability and reliability of fuel cell technology; for instance, FCEV may not achieve the same reliability characteristics as ICEs especially in some temperature and humidity conditions, or lower durability of FCEVs (29,000 to 75,000 miles) compared to ICEs (last on average up to 150,000 miles)
	• Reduction in greenhouse gas emissions	• Safety concern for hydrogen tank and fueling system
	• Zero tailpipe emissions (for hydrogen as fuel and water is the only product)	• Slight increase in refueling time for hydrogen (about 5–10 min) compared to traditional vehicles' fueling time (about 2–3 min)
	• Lower noise and better smoothness	
	• Few maintenance requirements	
	• Modularity (FCEVs' efficiency is almost constant with FCEV's system rated power)	
	• Better running cost and maintenance	
	• Reduction in gasoline and diesel fuels consumption	
	• Fuel required for FCEVs can be obtained through both renewable and nonrenewable feedstocks	

**Figure 4:**
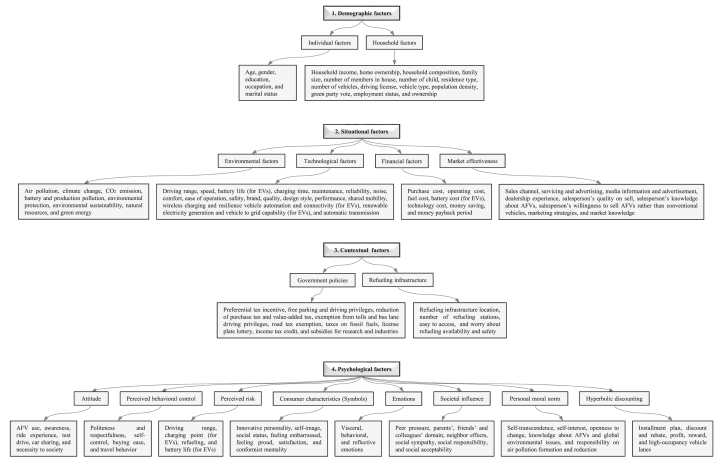
Identification and categorization of the main factors affecting AFVs' actual adoption (the figure is adapted from Ref. [71] with permission from Elsevier (License Number: 5100040378876)).

In the third step, those factors that have effects on the purchase intention of AFVs by consumers were identified and categorized (as shown in Figure 4). Then, after overviewing the latest studies (from 2015 to 2021), we have suggested that although the technical and financial factors (especially purchase cost) are the main barriers to the adoption of AFVs, there could be two other very important and fundamental points that might cause increases in the demand and adoption of AFVs. These two points include the car salespeople's and people's knowledge on AFVs and the environmental issues, and their encouragement by governments and policymakers to accept and use AFVs. In the fourth step, several recommendations to governments and policymakers have been presented to increase the actual adoption rate of AFVs. Finally, the conclusions have been drawn.

It is worth mentioning that the data required for this review was taken from several search engines and databases like Web of Science, Scopus, Google Scholar, Google, etc. The initial number of publications was about 4000, including papers, reports, and websites, while it was reduced to about 350 after filtering their publication dates (2015–2021) and checking their titles, abstracts, and summaries. Then, we carefully read these 350 publications and the data related and required for this review was obtained accordingly.

## 3 Types of current AFVs available in the global transportation fleets

The common AFVs which have the potential to apply for the transportation sector at the present and in the near future (say 2050) include flexible-fuel, gas, electric, hybrid electric, and fuel cell electric vehicles. A brief description of these vehicles with their pros and cons is presented in this section, which is necessary to understand why some AFs are preferred for use by people, but some of them are not.

### 3.1 Flexible-fuel vehicle (FFV)

Flexible-fuel vehicle (colloquially called flex-fuel vehicle) has almost the same internal combustion engine as the conventional vehicle (gasoline or diesel), but the main difference is that the FFV is designed to run on more than one type of fuel. In FFV, the AFs are blended (mixed) with conventional fuel (gasoline or diesel). The most common AFs used for FFVs are ethanol (mostly up to 85% by volume) for gasoline vehicles [26] and biodiesel (mostly up to 20% by volume) for diesel vehicles [27]. It is noticeable that the term flexible-fuel mostly refers to ethanol-gasoline fuel (not for biodiesel-diesel fuel or other biofuels blend) on the market and literature. The main fueling system of FFVs is almost the same as that for the conventional vehicles, which consists of only one fueling system (no extra fueling system is required); while there are needs for modifications (except for using 20% or lower biodiesel percentage [28], and 10% or lower ethanol percentage [26]) to the fuel tank, fuel pump, and fuel injection system due to differences in the physicochemical properties of AFs compared to gasoline or diesel fuel. In addition, the electronic control unit is also needed to be calibrated based on the type and concentration of alternative fuel used [26]. Generally, 20% or lower biodiesel percentage, and 10% or lower ethanol percentage in the blend can be used in the current diesel and gasoline engines, respectively without the needs for special modifications [26, 28].

In addition to ethanol and biodiesel, there are emerging AFs, including biobutanol, dimethyl ether, methanol, and renewable hydrocarbon biofuels (renewable gasoline or diesel) that are either under development or already have been developed, and may be available and commercialized in the transportation sector in the future to increase energy security, drop emissions, and improve vehicle performance [29].

#### 3.1.1 Ethanol

Ethanol, or ethyl alcohol, is a clear and colorless liquid that can be used as a fuel in gasoline and diesel vehicles. Ethanol with different blending percentages with gasoline (mostly up to 85% of ethanol by volume [1, 6, 30, 31]) in gasoline vehicles is commercialized and available on the market. But it is almost in the laboratory scale (almost not widely commercialized) in diesel engines called E-diesel (mostly 10–15% of ethanol) due to its huge problems like lower engine performance, lower flash point (more flammable and increasing explosions risk in traffic incidents), lubrication problem for fuel-injection, and difficulty in achieving a stable blend compared to those of diesel vehicles [26]. It is noticeable that ethanol can also be used in its neat form (100% ethanol) if a proper infrastructure for the engine is applied (in Brazil [31], as an example). Up to today's technologies, ethanol can be produced through fermentation and distillation of edible biomass feedstocks such as corn grain, sugar cane, sugar beet, etc. (known as 1^st^ generation ethanol) and non-edible biomass feedstocks such as wood chips, agricultural residues, cellulose, etc. (known as 2^nd^ generation ethanol) [32]. The contribution of the 1^st^ generation ethanol is much higher than the 2^nd^ generation ethanol on the current market.

#### 3.1.2 Biodiesel

Biodiesel can be produced from various feedstocks through the transesterification process. Up to the present, the feedstocks for biodiesel production are categorized based on the production generation as edible biomass feedstocks such as soybean oil, rapeseed oil, coconut oil, corn oil, palm oil, rice oil, etc. (1^st^ generation), non-edible biomass feedstocks such as jatropha oil, neem oil, karanja oil, rubber seed oil, calophyllum inophyllum oil, etc. (2^nd^ generation), micro algae, waste cooking oil, and animal fat (3^rd^ generation), and engineered photosynthetic microorganisms and synthetic cell (4^th^ generation, almost in conceptual/laboratory stage) [33, 34]. Commonly, biodiesel is mixed with diesel to use in compression ignition (CI) engines up to a certain level (mostly 20% of biodiesel), because there are no needs for modifications of current engines to use 20% or lower biodiesel percentage in the blend. However, biodiesel can be also used in CI engines in its neat form; while there are needs for engine modifications, particularly in the fueling system [28].

### 3.2 Gas vehicles

Gas vehicles available on the market are mostly using natural gas, liquefied petroleum gas (LPG), and hydrogen as a fuel. Natural gas for vehicles is used in the form of compressed natural gas (CNG) and liquefied natural gas (LNG). Natural gas consists of mostly methane (over 90%) and a small concentration of other hydrocarbons, which can be obtained from wells during crude oil production (non-renewable, which is available globally) or biogas (called renewable natural gas or biomethane, which is not widely commercialized). Natural gas has been used worldwide to generate heat for homes and industries for a long time, and it has been selected to be an alternative fuel in transportation fleets since the past decades. There are currently three types of natural gas vehicles (NGVs), including dedicated (run vehicle only on natural gas), bi-fuel (two separate fueling systems to run vehicle on either natural gas or gasoline/diesel), and dual-fuel vehicles (run vehicle on natural gas but use diesel fuel for ignition assistance) [26, 35].

LPG, also known as propane or propane autogas, is a clean fuel, compared to diesel and diesel fuels, which has been selected to be an alternative fuel in transportation fleets from the past decades, while it has been used as an energy source for homes and industries since many years ago. LPG mostly consists of propane (over 90%) with small concentrations of other gases, and it is produced as a by-product of crude oil refining and natural gas processing [29]. Similar to the natural gas vehicle, LPG vehicle also can run on LPG only (type dedicated vehicle), or switching between LPG and conventional fuels like gasoline (type bi-fuel vehicle) [36, 37].

Hydrogen can be extracted from fossil sources like oil and natural gas or from non-fossil sources like biomass and water through electrolysis, while currently, fossil fuel is the major source for hydrogen production. It can be used as an energy source for vehicles, including internal combustion engine (ICE) vehicles and fuel cell vehicles. However, currently, hydrogen as an energy source for fuel cell vehicle is much more applicate than that in the form of ICE vehicle, due to higher efficiency and zero tailpipe emissions of fuel cell vehicle [26, 29] (discussed in next sections).

It is noticeable that according to projection, after gasoline and diesel fuels, natural gas along with propane will be counted as the world's third most common transportation fuel by 2050 (currently about 23 million natural gas vehicles are available worldwide), due to their advantages such as availability, clean-burning qualities, high-energy density, and relatively low fuel cost for many countries [1, 29].

### 3.3 Fuel cell electric vehicles (FCEVs)

In FCEVs, the fuel cell generates the energy required for propelling the drive train and moving the vehicles. Fuel cell is an electrochemical device that converts the chemical energy of a fuel (mostly hydrogen, while hydrocarbons and alcohols can also be used) and an oxidizing agent (often oxygen) into electricity and heat [38]. Compared with conventional vehicles (gasoline and diesel) which have harmful tailpipe emissions, the product of hydrogen and oxygen reaction in FCEVs is a clean and environmentally friendly material which is water.

### 3.4 Electric vehicles (EVs)

EVs use electric motors or traction motors to propel the vehicles. EVs also can be combined with ICEs. There are generally three types of EVs available on the market, including battery electric vehicles (BEVs), hybrid electric vehicles (HEVs), and plug-in hybrid electric vehicles (PHEVs). The global EV fleet is about 8.5 million (more than 500 thousand buses, more than 400 thousand electric delivery vans and trucks and the rest counted for passenger vehicles) currently (2019–2020) and it will be about 116 million [39] (or EVs sales reach 23 million and the stock will be more than 130 million, excluding two/three-wheelers according to the IEA New Policies Scenario [40]) in 2030, while global vehicle fleet is about 1.2 billion in nowadays (2019–2020) and it will be about more than 1.4 billion in 2030 on the road [39]. These projections show that despite more developed technologies on EVs causing a reduction in their price and an increase in their performance and mileage, and infrastructure, just less than 9% of the global vehicle fleet will use EVs in the future (say 2030), which it is only a little more contribution than that of EVs in global vehicle fleet in 2020 (contribution of about 7%).

BEVs (also called pure EVs, only-EVs or all-EVs) have only electric motors (or traction motors), while the other EVs consist of both electric motors (or traction motors) and ICEs to propel the vehicle. BEVs receive all the electricity required for charging their batteries and propel the vehicle from an external electric source, such as through a wall socket or a charging station. HEVs (also called conventional hybrids) receive their electricity only from the ICEs (smaller than traditional ICEs). If the batteries are low, the ICEs work as secondary means of propulsion system to propel the vehicle as well as charging the batteries [26, 29].

PHEVs have the same working principle as HEVs, while the only difference is that PHEVs can also receive their electricity from an external electric source (like a wall socket or a charging station). The size of batteries for PHEVs is bigger than those of HEVs, resulting in achieving more travel distance on electricity only. If PHEVs do not use an external electric source, their fuel economy will be almost the same as similarly sized HEVs. There are two kinds of PHEVs, including series plug-in hybrids (also called extended range electric vehicles (EREVs) and parallel (blended) plug-in hybrids. In series plug-in hybrids, the electric motor only turns the wheels, and ICEs work only to generate electricity. However, in parallel plug-in hybrids both electric motors and ICEs are connected to the wheels and propel the vehicle. It is noticeable that in addition to the use of an external electric source and ICEs to charge the EVs’ batteries, generation of electricity from the energy harnessed called “regenerative braking” during the braking can also be used [26, 29].

## 4 Benefits and drawbacks of AFVs

The benefits and drawbacks of AFVs due to their different feedstocks, production processes, and fuel properties, compared to the gasoline and diesel vehicles, make them a favorable or unfavorable resource for the global transportation sector, respectively. These benefits and drawbacks are listed in Table 1.

## 5 Factors affecting people's purchase intention on AFVs

**Figure 5:**
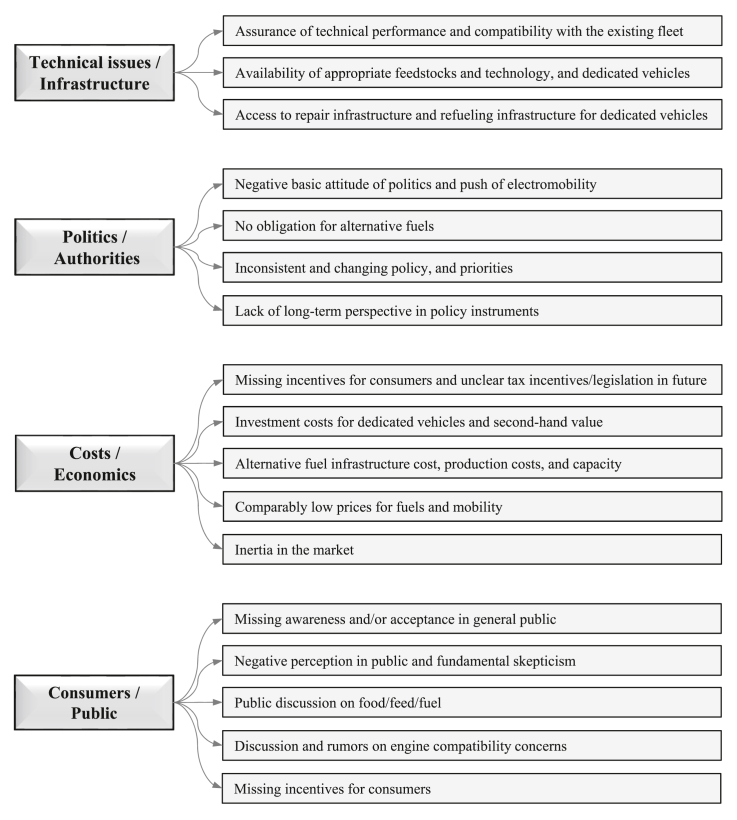
Main barriers to introduce the AFVs to the market in the form of either purchasing new AFVs or converting gasoline/diesel vehicles to them. Note: the figure is adapted from Ref. [13] with permission from IEA Bioenergy (the report was published in November 2020).

**Figure 6:**
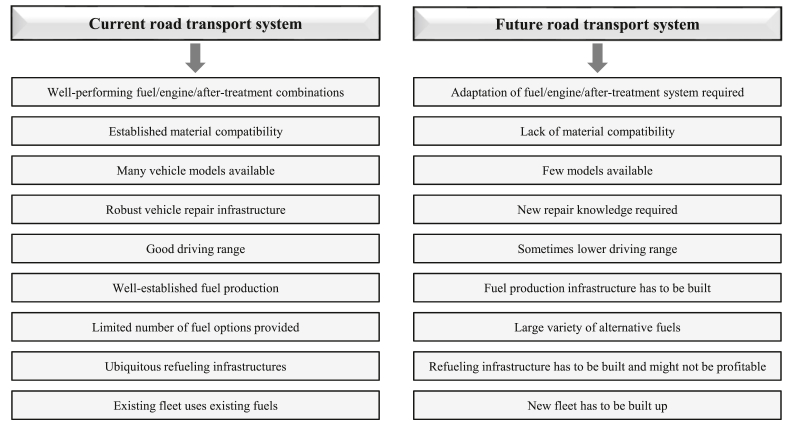
Features of the current road transport system available for conventional vehicles and AFVs, and the future road transport system (projected to 2050) available for new AFVs or converting gasoline/diesel vehicles to alternative fuel vehicles. Note: the figure is adapted from Ref. [13] with permission from IEA Bioenergy (the report was published in November 2020).

Vehicle, fuel and upfront cost, infrastructure (charging or refueling stations), fuel taxation, vehicles performance, subsidies from governments to use AFVs, environmental concern, awareness of responsibility for environmental protection, public acceptance for AFVs, knowledge about global air pollution issue and AFVs, type of AFVs, among others (as shown in Figure 4, data is taken from the latest review papers, including Refs. [54, 58, 59, 60, 61, 62, 63, 64, 65, 66, 67, 68, 69, 70, 71, 72, 73, 74, 75, 76, 77, 78, 79, 80, 81, 82, 83]), have a strong relationship with vehicle purchase intention and willingness-to-buy (or willingness-to-pay) by consumers, but the intensity of each parameter varies for people in different countries. In addition, the major current barriers to introduce the AFVs to the market in the form of either purchasing new AFVs or converting gasoline/diesel vehicles to them. The features of the current and the future road transport systems are presented in Figure 5 and Figure 6, respectively, according to the latest IEA Bioenergy report in November 2020 [13]. The barriers vary from country to country, depending on several factors mainly policy, regulatory framework, public acceptance, economic competitiveness, infrastructure, technical and commercial maturity, and availability of appropriate feedstocks; therefore, every country needs/has to find its own optimum alternative fuels [13]. Most of these barriers are related to the technical and economical factors which can be solved only by improvement in technologies year by year, but some of them which are also very important can be solved by the current technologies. Those include public knowledge, opinion, and acceptance of alternative fuels. At the beginning of AFVs adoption in Austria, as an example, the introduction of E10 vehicles was stopped several weeks before their market entry, mostly due to the public discussion on food/feed vs. fuel and engine compatibility, although there was suitable technology for E10. Because the people had no suitable knowledge about the performance of E10 vehicles and worried about the ethanol feedstocks. In another example, after the successful introduction of CNG vehicles into the market in Austria, the number of CNG vehicles and gas stations were decreased due to the missing acceptance of the general public on CNG vehicles [13].

Thus, to succeed in the adoption of AFVs in the global transportation fleets, the application of AFVs needs to fulfill the requirements of the innovation-decision process, because they can be counted as new and innovated materials. It is well known that according to the innovation-decision process, individuals need to pass through five steps for adopting or rejecting any innovations or changes (e.g., using AFVs). The five steps include 1) knowledge, 2) persuasion, 3) decision, 4) implementation, and 5) confirmation [84]. We can see that knowledge/information about the product is the first and the most important factor in the decision process, while in adopting AFVs, we can see from Table 2 that although the AFVs are available on the market for many years, many people around the world, even in the developed countries and even in the present decade, have no/less/old/wrong knowledge about the advantages and disadvantages of AFVs in comparison with those of gasoline and diesel vehicles. Thus, most of these people reject these vehicles for use, even when their important parameters such as purchase price, operating cost, driving range, and fuel availability be the same (or close) as gasoline or diesel vehicles.

**Table 2:** Effect of car salespeople's and people's knowledge on AFVs and the environmental issues, and their encouragement by governments and policymakers compared with the technical factors on people's purchase intention for AFVs.

Country/region	Type of AFVs	Samples	Parameters investigated	Findings	Recommendations to rise in willing to use AFVs	Year and reference
Germany, India, Japan, Sweden, UK, and USA	Electric, hybrid, and biofuel vehicles	6033 participants (about 1000 vehicle consumers per each country)	Consumer intentions for AFVs	Electric and hybrid vehicles had more intention to be used as alternative vehicles rather than biofuel vehicle in almost all countries; Hybrid and EVs had more intention to be used rather than gasoline vehicles due to the environmental concern in Germany, India, Japan, but not for other countries; Indian participates had the highest concern about the environmental issues (82% of participates), while the highest number of participants (about 41%) would be embarrassed if their family and friends know about their concern about the environment and having an environmentally friendly lifestyle; but in contrast, Japanese participants had the lowest concern about the environmental issues (33%) as well as lowest embarrassing rate (11%) among all countries investigated; other countries had almost similar trends to each other	In some cases, the intention of using biofuel vehicles was less than gasoline vehicles in almost all countries investigated, therefore, there is a need for more information about the effect of biofuel vehicles on the vehicle performance and the environment; In addition, there is a need for explaining to people that the environmental issue is a big challenge nowadays, and concerning about it or having an environmentally friendly lifestyle will not be an embarrassing opinion	2020 [88]
France, Germany, Italy, Spain, Poland, and UK	Electric vehicles	3723 participants (about 600 cases per each country)	Effect of vehicle price reduction, increase in driving range, recharging time, possibility of recharging at home, and increase in maximum speed on vehicle purchase intention	Price reduction had the highest effect followed by improvement in the driving range and the possibility of recharging at home on vehicle purchase intention, while maximum speed had the lowest effect; Huge concerns about vehicle price could be due to lack of people's knowledge about the long-term benefits of EVs on fuel saving and emissions reduction; High income and high-educated people had more intention to purchase EVs compared to low income and less-educated people; More than half of participants in each country (especially Poland with 71%) had no useful knowledge about EVs (especially on the charging time, fuel economy, and availability of fast charging method), while most of participants only knew that EVs are expensive, silent and safe with no tailpipe emissions (similar to a review paper [52] which found many European people have lack of sufficient information about EVs)	There are needs for engaging in technological development from governments and manufacturers sides to reduce the electric vehicle price as well as improvement in the quality of batteries to increase in the driving range; Governments and public campaigns can provide financial incentives and more information and knowledge for people about the EVs and their possible long-term benefits on fuel saving and emissions reductions	2018 [89]
Denmark, Finland, Iceland, Norway, and Sweden	Electric vehicles	Data from 126 mystery shopping experiences at 82 car dealerships across five countries	Analyze the effect of direction and level of orientation by sales personnel to customers to or away from EVs	It was found that most of salespeople were dismissive of EVs, misinformed customers on the specifications of vehicles, neglected EVs from the sales conversation, and even strongly oriented customers towards purchasing gasoline/diesel vehicles, therefore, just 8.8% of the mystery shopping encounters resulted in the customers having preferred EVs as an option for their next car purchase over gasoline/diesel engines	Car salespeople's technological orientation, their willingness to sell EVs over gasoline/diesel vehicles, and displayed knowledge of EVs can be counted as the main contributors to increase in EV purchase intentions, which could be supported and provided by governments, policymakers, and industries; Also, policy and business strategies that have significant effects on addressing the barriers at the point of sale are required to rise the EV adoption rate	2018 [87]
China, Brazil, and Russia	Electric vehicles	2806 participants (China = 1078, Brazil = 929, and Russia = 799)	Consumer intentions for AFVs	Chinese people had more intention to purchase EVs than Russian and Brazilian people due to their wider social network and more information about EVs from somebodies using EVs, while concern about pollution and charging infrastructure had low impacts	Since information and knowledge about EVs have a significant impact on the increase in people's purchase intention, therefore, governments need to invest more on these parts; In addition, there is a need for the increase in people's knowledge about the current environmental issue (especially in China) and the positive effect of EVs on it	2018 [90]
China and USA	Battery electric, hybrid electric, and plug-in hybrid electric vehicles	832 participants (China = 448 and USA = 384)	Effect of personal experience, attitudes, and knowledge about driving and EVs, environmental attitudes, and subsidies on willingness-to-pay for EVs	Chinese people had more willingness-to-pay for EVs (especially battery electric vehicles) than American people due to their more concern on environmental issues	Since environmental issues are concerned, governments need to invest more on these parts to educate people about the positive effect of clean-energy vehicles like EVs on the reductions of GHG and air pollution	2015 [91]
Canada	Electric vehicles	95 mystery shopping experiences from twenty trained mystery shoppers at 24 EV-certified dealerships in Ontario	Effect of availability of EVs at the dealerships and salespeople's knowledge confidence, and enthusiasm about EVs specifications and subsidies on customer intentions for EVs	It was found that the availability of EVs at the dealership could be a common advantage for EVs adoption, while there are some significant barriers from the salespeople's side, for example, some of them have no information or misinformation about the EVs specifications (6% of responses) and subsidies (34% of responses), and some of them did not inform the customers about the availability of Ontario subsidies on EV purchase cost (34% of responses) and EV charges (71% of responses)	It is suggested that government and dealerships can be partner to increase the salespeople's knowledge about the EVs specifications and subsidies by training them through online resources and/or in-person method on a regular time (e.g., every six months), as well as providing sufficient EVs at the dealerships to view or test-drive by costumers with shortening the time required (3–4 month waiting periods) for receiving some EV models after ordering	2017 [92]
China	Electric vehicles	1087 participants (from 29 provinces and 206 cities), while 95% had 1 or 2 cars	Effect of psychological attributes (attitude, subjective norms, and perceived behavioral control) and policy attributes (purchase subsidies, license plate control, preferential usage, and preferential driving) on vehicle purchase intention	It was found that there was a necessary need for participating at least one psychological attribute to have high purchase intention, even if government implemented purchase subsidies, the joint absences of psychological attributes caused low purchase intention by people	It is suggested that government and policymakers need to focus more on the human behaviors' side rather than the only subsidies' side	2021 [93]
China	Hydrogen fuel cell electric vehicles	1072 participants (people who had one vehicle)	Effect of purchase price, refueling time, driving range, fuel cost, emissions reduction, and refueling accessibility on vehicle purchase intention	Participants agreed to pay more money for FCEVs if they have higher driving range (about 200 km) or lower emissions (even -20%) compared to those of gasoline vehicles, while the other parameters (refueling time, refueling accessibility, purchase price, and fuel cost) had less effect than driving range and emission reduction on vehicle purchase intention	There are needs for improvement in driving range of FCEVs as well as informing people about the strong effect of FCEVs on emissions reduction; Government subsidies can help to reduce the purchase and fuel costs for FCEVs; Government can focus on specific vehicle consumers (e.g., well-educated people, long-distance travelers, or consumers who have concerns about the driving range) compared to regular vehicle consumers, because those people have more willing to purchase FCEVs than regular vehicle consumers	2020 [56]
China	Electric vehicles	651 participants (people from 16 administrative regions in Beijing)	Effect of vehicle price, vehicle usage (operating cost, driving range and infrastructure), social influence (media, advertisements, neighbors, and friends), environmental awareness, purchase policies (subsidies, tax incentives and license fee exemption) and usage policies (free parking, bus lane access, and toll exemptions) on vehicle purchase intention	Vehicle price (32.3%) and vehicle usage (28.1%) had the highest effect, while the environmental awareness (9.6%) and usage policies (7.8%) had the lowest effect on vehicle purchase intention	Since vehicle price and vehicle usage are important factors to purchase EVs, therefore, government should pay more attention to them during making policies, and financial incentives and more investment on charging infrastructures also can be useful to increase in EV purchase intention; There is a need for the increase in people's knowledge about the current environmental issue (especially in China) and the positive effect of EVs on it	2019 [94]
China	Electric vehicles	320 participants (consumers at 40 Auto shops in 10 cities)	Effect of perceived usefulness, attitude towards EVs, perceived risk, knowledge about EVs, and financial incentive policy on vehicle purchase intention	All parameters (except for financial incentive policy) had significant effects on vehicle purchase intention, while knowledge about EVs had the highest effect	Most of the Chinese consumers agree to purchase EVs when they have sufficient knowledge and information about them (usage cost, charging time, driving range, etc.), and they believe that financial incentive policy will not be important as knowledge about EVs; Lacking knowledge about EVs and perceiving high risk can be counted as the psychological barriers to the acceptance of EVs by Chinese consumers, therefore, government needs to increase the people's knowledge about EVs rather than just the increase in the financial subsidies	2018 [95]
China	Electric vehicles	324 participants (consumers at several Auto shops in 10 cities)	Effect of policy measures (financial incentive, information provision, and convenience) and environmental concern on vehicle purchase intention	All four parameters had a significant positive effect on vehicle purchase intention, while convenience policy measure and environmental concern had the highest and the lowest impacts, respectively	Government can provide more convenience policy measures (e.g., building more charging infrastructural facilities and parking spaces, etc.) as well as increase the people's knowledge about the current environmental issue and the positive effect of EVs on it	2017 [96]
China	Battery electric, hybrid electric, and plug-in hybrid electric vehicles	655 Chinese college students	Effect of environmental attitudes, perceived behavioral control, subjective and descriptive norms, and renewable energy knowledge on the adoption of EVs	All five parameters had a significant positive effect on adoption of EVs, while environmental attitudes had the highest impact, and the others have very close effects (almost half of the environmental attitudes)	Since Chinese college students have concerns about the environmental issues, by far compared to the other factors, they will buy EVs, therefore, government can educate people and vehicles' buyers about the effect of EVs on air pollution reduction compared to traditional vehicles, as well as giving subsidies to research and industries to improve in the EVs' technologies	2016 [97]
Finland	Biofuels	90 participants (70% of participants had traditional cars)	Public acceptance and knowledge about biofuels	• 60% of participants were willing to use biofuels, but lack of information about biofuels prevented them to use biofuels; • 60% of participants agreed to pay more biofuel cost than gasoline and diesel fuels (at least 5% more) due to the environmental concern, if they are available similar to gasoline and diesel fuels; • Some participants had wrong information, e.g., 23 and 97% of them thought that biofuels (from biomass) are non-renewable and zero GHG emitter, respectively; • 50% of participants did not agree to use biofuels because thought they have a direct effect on food price	• Government can provide further information about biofuels, reduce the biofuels cost and use non-edible feedstock for biofuels production to encourage people to use biofuels; • 60% of participants mentioned that reducing the biofuel cost would be the best solution from the government's side	2017 [98]
Germany	flex-fuel vehicles (dimethyl ether (or blend of diesel with oxymethylene dimethyl ethers)	256 participants (drivers of gasoline, diesel, hybrid, gas or EVs who had annual mileage experience of at least 5000 km)	Effect of fuel cost and availability, driving range, pollutant emissions, and usage requirements on using AFs	Fuel costs (+0, +10 or +20 Euro cents/liter compared to diesel) followed by fuel availability (10, 50 or 100% of refueling stations) had the highest effect on selecting AFs, while pollutant emissions (-10, -30 or -60% reductions compared to diesel) had the lowest impact on selecting AFs	Reduction in fuel price (or even tax reductions or subsidies), providing more refueling stations as well as public information about them can increase the intention of drivers to use AFs; There is a need for educating people and drivers about the current environmental issues and the effect of using alternative fuels on them (reduction in air pollution and GHG emissions)	2019 [99]
India	Electric vehicles	Data was taken from 79 articles during 2009–2019	Effect of seven factors, including technological, social, cultural, economic, political, geographical, and environmental with 67 variables on vehicle purchase intention by Indian people	Technological (range, charging time, fuel economy, etc.) with 0.23902 category weight followed by environmental (GHG emission, global warming, air quality, noise, etc.) with 0.20924 category weight are the most critical factors, while economic (purchase cost, fuel price, operating and maintenance costs, etc.) with only 0.16432 category weight had the third important place and geographical (fossil fuel availability, renewable electricity, and raw material availability) had the lowest impact with 0.04491 category weight	Since Indian people have concerns about the technological and environmental issues, by far compared to the other factors, and they will buy EVs even their purchase and fuel price, etc. (from economic category) be high and even government subsidy and tax exemption (from political category) be less, therefore, government can educate people and vehicles' buyers about the effect of EVs on air pollution reduction compared to traditional vehicles, as well as giving subsidies to research and industries to improve the EVs' technologies	2021 [100]
India	Electric vehicles	228 participants (EVs' buyers)	Effect of performance features, financial advantages, environmental concerns, social influence, cost of ownership, and infrastructure support on vehicle purchase intention	The environmental concerns followed by performance features had the highest impact on vehicle purchase intention, while both cost of ownership (vehicle price) and infrastructure support (charging stations) had no significant effect on vehicle purchase intention	Since participants have concerns about the environmental issues, as well as concerning about EVs performance, and they will buy EVs even their price be high, therefore, government can educate people and vehicles' buyers about the effect of EVs on air pollution reduction and their performance compared to traditional vehicles	2019 [101]
India	Electric vehicles	233 participants (service and business class people who used cars almost every day)	Effect of environmental concern, vehicle and fuel cost, vehicle comfort, trust, technology, infrastructure and social acceptance on vehicle purchase intention	The environmental concern and technology (vehicle speed and efficiency, and traveling distance) were the first and second important factors followed by vehicle and fuel cost, while social acceptance was the least parameter; urban respondents had more social acceptability than rural respondents due to lack of information about EVs	The participants have concerns about the environmental issues, so government should provide sufficient facilities for EVs as well as further information (especially in the rural area) about the performance of EVs to increase in social acceptance on using EVs	2018 [102]
Iran	Natural gas, liquid petroleum gas, biodiesel, biogas, ethanol-gasoline (E85), methanol-gasoline (M85), and hydrogen	Information and models were obtained from reviewing several papers	Effect of financial-, technical-, social- and policy-related parameters on adoption of AFs for light-duty vehicles	Petroleum fuels counted to be the main source, mostly due to higher cost and lack of infrastructure, investment, and knowledge about AVs, insufficient social and environmental policies from government and policymakers, insufficient training and social acceptance, along with insufficient plans for biofuels production; Natural gas and liquid petroleum gas were the most suitable AFs due to their more availability and environmental benefit, and lower price among all the AVs investigated	Iranians have concerns about the current environmental issues, while their insufficient knowledge and technical and financial barriers (particularly higher price) on AFVs need to be addressed by government and policymakers for increase in the adoption rate of AFVs	2017 [78]
Japan	Electric vehicles	106446 participants (non-EV owners)	Effect of environmental awareness and evaluation of EVs (vehicle performance, price, infrastructure, and driving range) on vehicle purchase intention	Evaluation of EVs had more effect than the environmental awareness to purchase EVs; Only 5.5% of participants had serious intention to purchase EVs, while 94.5% of participants had low or no consideration to purchase EVs at the current environment and technology conditions which was similar to [103] in the USA with a serious intention of only 3.5% of participants for PEVs; Only about 15 and 45% of participants agreed that awareness about the current environmental issues is very important and somewhat important, respectively	Government should not only inform people the performance of EVs, they also should inform people about the effect of EVs on the reduction of air pollution and GHG emissions (especially CO_2_); Using separate advertisements about the environment and EV's performance will be more useful for encouraging females and males, respectively to purchase EVs	2019 [104]
Macau	Battery electric vehicles	308 participants (those divers who had their own traditional cars)	Effect of environmental concern, and economic benefit and performance of BEV on purchase intention to purchase BEV	All three parameters had a significant positive effect on purchase intention to purchase BEV in 3 years, while economic benefit (fuel cost and saving) of BEV and performance of BEV had the highest and lowest impact, respectively	Since the BEV economic benefit is more important than the environmental concern and BEV performance for participants, thus, there are needs for actions by government for reducing the BEV operating cost (for fuel saving) and giving subsidies (e.g., tax exemption, free parking, free public charging using solar powered charging facilities, etc.) as well as an increase in people's knowledge about the current environmental issues and the effect of BEV on air pollution reduction (for adults and students in schools), to rise in BEV purchase intention by people	2015 [105]
Malaysia	Hybrid vehicle	380 participants (students, staff, managers, and lecturers at two universities in Malaysia)	Effect of price sensitivity, environmental awareness, green perceived value, and green trust on vehicle purchase intention	Among all five parameters investigated, only price sensitivity and green trust had a significant effect on hybrid vehicle purchase intention	There are needs for the increase in people's knowledge about the environmental issues as well as reducing the hybrid vehicles' price to rise in hybrid vehicle purchase intention by people	2017 [106]
Malaysia	Natural gas vehicle	152 participants (gasoline vehicle owners and drivers)	Effect of refueling station and time, money payback period and fuel price on consumers intention for converting gasoline vehicle to NGV	Only 34.2% of participants were willing to change their gasoline vehicle to NGV, while they were concerned about NGV safety, sustainability, performance, speed, engine durability, available fueling conversion kits in country, government subsidy, and refueling stations	Little knowledge of participants about NGV and its advantages coupled with some infrastructure issues for NGV was the main issues for the low intention of participants to use NGV; therefore, education people about NGV and effect of GHG on the environment and human health via media, giving some subsidies to people, and increase in the number of refueling stations by government could be the solutions	2015 [107]
Malaysia	Hybrid vehicle	121 participants (those people who held driving license and owned no hybrid vehicle)	Effect of environmental attitude, social influence, and awareness of responsibility on vehicle purchase intention	All three parameters investigated had a significant positive effect on hybrid vehicle purchase intention by people; 96% of participants were willing to buy hybrid vehicle due to their concern about the environmental issue, but if government could waive the import and excise duty for hybrid vehicles	Since people have concerns about the environmental issues and also aware of their responsibility on air pollution reduction, therefore, government can encourage people to use hybrid vehicles through these parameters via media, as well as waiving the import and excise duty for hybrid vehicles	2015 [86]
South Korea	flex-fuel vehicles (ethanol)	471 participants (owners of gasoline, diesel, or LPG vehicle)	Willingness to pay for the second-generation bioethanol (lignocellulosic) for vehicles	72.6 and 14.2% of participants would purchase bioethanol at the current market price (more expensive than gasoline) and at a premium price, respectively, while 8.1% of participants would not purchase it even with a discount; Participants who received positive information about environmental advantages of bioethanol had more willing to pay than those who did not receive any additional information on bioethanol; Female and high-educated people had more willing to pay for bioethanol compared to male and less-educated people	There is a need for the increase in people's knowledge about the biofuels, so government can inform people about lower greenhouse gas emissions, water consumption, and pesticide and fertilizer use for bioethanol production, and lower air pollution and GHG emissions of bioethanol combustion in vehicle; Educating people about renewable energy supply can have a positive influence on willing to pay for the advanced biofuels	2019 [108]
South Korea	Fuel cell electric vehicles	1000 participants (those people who were interested to purchase a vehicle)	Effect of fuel efficiency, fuel accessibility, air pollution and CO_2_ on willing to pay for vehicle	The participants had a deep interest on paying more money for FCEVs and placed significant values on the FCEVs' attributes; Reduction (1%) in air pollution and CO_2_ had the highest effect on willing of pay for FCEVs, while improvement in fuel accessibility (1%) had the lowest effect	Government subsidy can play a significant role for diffusion of FCEVs	2019 [109]
South Korea	Battery electric, hybrid electric (gasoline + battery), and fuel cell (hydrogen) electric vehicles	1049 participants, (740 drivers and 309 non-drivers)	Effect of purchase and fuel costs, accessibility of fueling stations, Subsidies, and environmental issue on consumers' preferences for AFVs	Drivers preferred order was hybrid vehicles, EVs, gasoline vehicles, FCVs, and diesel vehicles, and non-drivers had also a similar order except for a change in the order of hybrid vehicles and EVs; Hybrid vehicles followed by EVs were preferred over FCVs due to their higher infrastructure availability and lower prices; Both drivers and non-drivers had significant environmental concerns	Government and policymakers need to reduce the purchase cost and increase the infrastructure, while expanding more budget on purchase subsidies over infrastructure subsidies can have more impact on environmental improvement by increase in AFVs adoption; Since environmental issues are concerned, government and policymakers need to invest more on these parts to educate people about the positive effect of AFVs on the reductions of GHG and air pollution	2019 [110]
Sweden	flex-fuel vehicles (ethanol)	12 years of monthly Swedish data	Fuel choice and fuel demand elasticities	Majority of multifuel vehicles drivers would not use ethanol, even its price be the same as gasoline; Increase in gasoline tax would cause limited success in making drivers shift to ethanol	Non-price attributes have a significant role in consumer decision-making. For example, drivers think that ethanol will damage the engine (due to its corrosivity) even for engine with new technology, and it causes to freeze the overall fuel mixture in winter for any kind of ethanol. So government should educate them about ethanol, in which, new engine has almost no problem with run on ethanol, like Brazil which uses 100% ethanol for cars, and ethanol containing less than 1% water has significant resistance to freezing	2018 [24]
Thailand	Electric vehicles	50 participants (90% of participants had traditional cars)	Effect of finance, performance, infrastructure, market efficiency awareness, environmental impact, information awareness, and government support on vehicle purchase intention	56% of participants were willing to use EVs in the future, while the rest of participants would not use EVs due to their little knowledge about EVs; Infrastructure (charging stations) and financial (vehicle and fuel price, and operating and maintenance costs) factors were the most important parameters, while the environmental impact and information awareness were the least important parameters	In addition to increasing in the investments for financial and infrastructure sides, there is a need for educating people about the current environmental issue and knowledge about EVs	2018 [111]
UK	Electric vehicles	1347 participants (people who will and will not tend to purchase EVs)	Effect demographic (individual and household), technological (10 items), financial (purchase, recharging and maintenance costs), and environmental factors on EV adoption	Purchase cost, performance, maximum range, and environmental were the main barriers; Younger and educated people had more intention for EVs; Some people (especially those who had no consideration to purchase EVs) had a lake of knowledge about EVs	Besides the needs for reduction in purchase cost and increase in performance and maximum range which have a significant influence on the diffusion of EVs, there is a need for the increase in people's knowledge and convincing (especially for older and uneducated people who have less interest to purchase EVs) about EVs and their advantages over ICE vehicles	2021 [112]
UK	Electric vehicles	26,000 participants (drivers of ICE vehicles)	Effect of 19 EV's barriers on EV adoption	Purchase cost and availability of public charging stations were the main barriers (for more than 81% of participants) followed by long offset time required for EV's higher cost via fuel and taxation saving (68%), battery performance (65%), driving range (59%), uncertainty about maintenance and repair infrastructure (58%), and so on, while lack of general understanding about the advantages of using EVs had a significant effect (34%, ranked 14 out of 19 barriers) on selecting EVs	Besides the needs for reduction in purchase cost and increase in charging stations which have a significant influence on the diffusion of EVs, there is a need for the increase in people's knowledge and convincing (especially for older people who have less interest to use EVs compared to young people) about EVs and their advantages over ICE vehicles	2018 [17]
UK	Electric, hybrid, and hydrogen fuel cell vehicles	413 participants (most of them had more than two traditional vehicles)	Effect of knowledge and persuasion on the decision-making process to adopt AFVs	Most of the participants (about 84%) had a low level of knowledge on AFVs; Male participants with socio-economic concerns and a high level of education had more knowledge on AFVs compared to female participants; Most of the participants were not concerned about climate change and the wanted to continue with traditional vehicle technology; Most of the participants (especially old people) would not purchase AFV, mostly due to the lack of knowledge about them	There is a need for educating people about knowledge on AFVs, especially list of models available, running costs, charging facilities, vehicle grant; Arranging vehicle showcasing events for people to test-drive AFVs; Making the cost of AFVs more comparable with traditional vehicles	2018 [113]
USA	Hybrid, plug-in hybrid, electric, natural gas, and hydrogen vehicles	1545 participants (car owners)	Effect of purchase price, driving range, parking price, parking search time and operating costs on selecting vehicle	Purchase price followed by driving range had the highest effect, while parking search time and operating costs had the lowest effect on selecting vehicle; Most of participants (64%) selected gasoline vehicle as their next vehicle to purchase, while 32% of participants selected EVs and the remaining 4% participants selected other types of AFVs coupled with diesel vehicle; For EVs, people were concerned about their range, charge times, operating costs, and parking, while the time for finding parking was more important than parking price	Besides the needs for technology improvement to drop the purchase price or increase in driving range for EVs (as the most preferred AFVs), there are some recommendations to increase adoption chance of EVs, such as allocation of specific parking places for EV only, reduction in parking fee and availability of battery swaps at charging stations	2020 [114]
USA	Hybrid, plug-in hybrid, and battery electric vehicles	1052313 participants (data of 11 years of new vehicle buyer, 2005–2015)	Finding latent demand (purchase price, fuel economy, environmental friendliness, technical innovation) for AFVs adoption	Potential to secure of AFVs was about 11% of the US market in 2015, however, the actual market share was only one-third of that; Hybrid vehicles had more potential and actual share among AFVs; Purchase price and environmental friendliness had the highest and lowest impact on selecting vehicle, respectively	Since there is a chance to grow up of potential AFVs to three times their current market size, therefore, tightening corporate average fuel economy and GHG emission standards, zero emitter vehicles mandate, and combining supply-side with demand-side support policies (e.g., financial and non-financial incentives such as subsidies, waiving of registration fee, free parking or waiving toll road fee) will cause more adoption of AFVs	2020 [115]

Besides the no/less/old/wrong knowledge of some people about AFVs, Table 2 shows that those people who have the concern about vehicle purchase and fuel price, have almost no/very less concern about the environmental issues and air pollution, and they will not pay more money for purchasing AFVs. In contrast, those people who have a big concern about the environmental issues and air pollution, are willing to buy AFVs. However, the contribution of eco-friendly people is very less than that of economical people, resulting in less contribution of AFVs in the global transportation fleets compared to that of gasoline and diesel vehicles in recent years and even in the near future. Thus, we can see that there is a need for educating and engaging people and car salespeople about the current global environmental issues and air pollution, and the positive effect of AFVs on them, to encourage people to use AFVs [85, 86]. The recent data of those countries which have high contributions in adoption, publication, or attention about AFVs in the world, regarding the effect of car salespeople's and people's knowledge on AFVs and the environmental issues, and their encouragement by governments compared with the technical factors on people's purchase intention for AFVs, is summarized in Table 2.

It is worth mentioning that paying more attention to the influence of car salespeople on the sale side could result in an increase in the adoption rate of AFVs. Because people mostly purchase the vehicles from the auto shops, and salespeople’ guide can play a significant role in their decisions. As an example, according to the data from 126 mystery shopping experiences at 82 car dealerships in a recent study published by Nature Energy in 2018 [87], most of the salespeople in Denmark, Finland, Iceland, Norway, and Sweden were dismissive of EVs, misinformed customers on the specifications of vehicles, neglected EVs from the sales conversation (in 77% of visits having EVs available in the shops), and even strongly oriented customers towards purchasing gasoline/diesel vehicles (in 67% of visits having EVs available in the shops), therefore, just 8.8% of the mystery shopping encounters resulted in the customers having preferred EVs as an option for their next car purchase over gasoline/diesel engines. A conversation between a salesperson and a vehicle customer in that study is interesting. The salesperson initially told “we don't have any [EVs]…they are more expensive, so they are probably not worth it”. However, when the vehicle customer later pressed the topic of EVs, the salesperson acknowledged “oh yeah, that's true, I do have a 100% electric [vehicle]”, though he still completely disregarded EV vehicle as a viable alternative. Therefore, if vehicle customers receive no/wrong/bad/insufficient information from the salespeople, they will ignore AFVs in their choices. Therefore, Car salespeople's technological orientation, their willingness to sell EVs over gasoline/diesel vehicles, and displayed knowledge of EVs can be counted as the main contributors to the increase in EV purchase intentions, which could be supported and provided by governments, policymakers, and industries.

## 6 Recommendations

**Figure 7:**
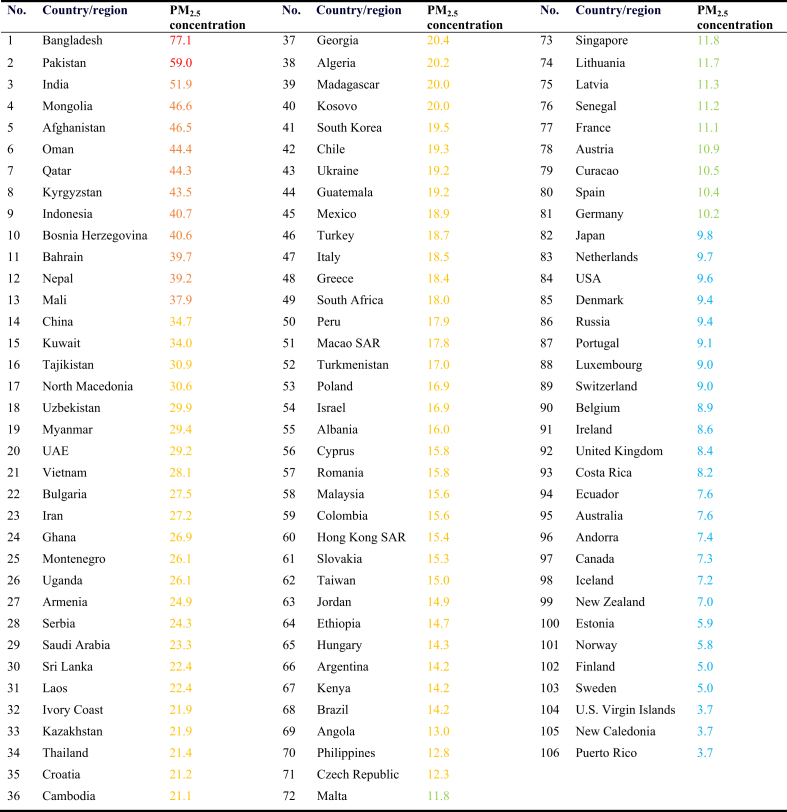
World country/region ranking for PM_2.5_ concentration, arranged by annual average PM_2.5_ concentration (μg/m^3^) and weighted by population in 2020 (the table is adapted from Ref. [14] with permission from IQAir). Note: to minimize the risk of the impact of PM_2.5_ on human health, WHO has set a guideline, since 2005, for the annual average PM_2.5_ concentration at 10 μg/m^3^ [14,116,118].

**Figure 8:**
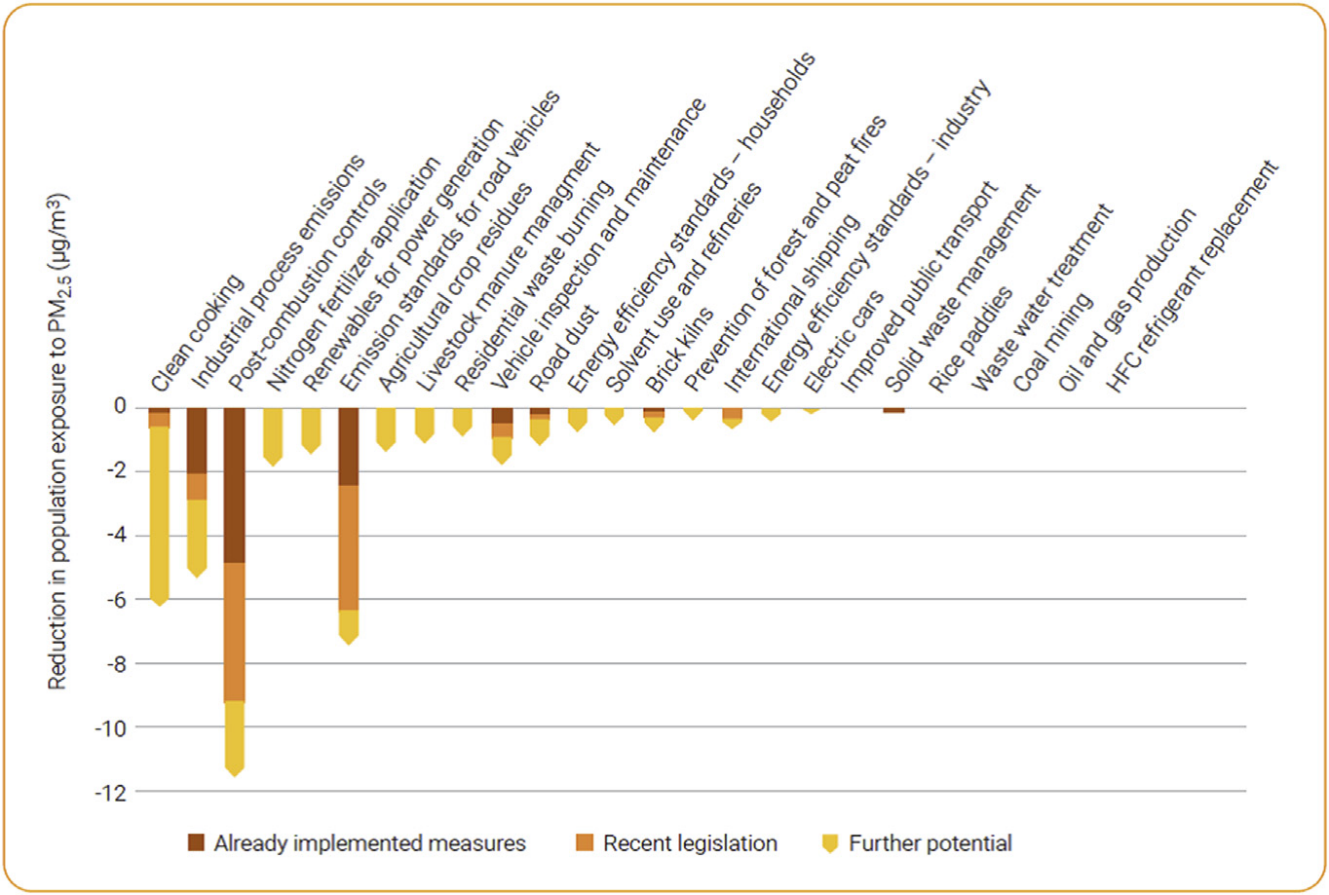
Impacts on population-weighted exposure to PM_2.5_ in 2030 from the implementation of 25 clean air measurements, ranked by further potential in Asia (the figure is reprinted from Ref. [119] with permission from UNEP).

It is recommended to governments and policymakers, encouraging the vehicles' users (including vehicles' owners and drivers as well as their families) to purchase AFVs, via several methods as discussed later, can be more effective than waiting for more rising in technologies for the production of easier and cheaper AFs in the future. The demand is a lost part of the vehicles market's chain which governments need to focus on that to succeed in rising the number of AFVs in the transportation sector and drop in air pollution. In the past and present (and in the future for sure) we have suitable and promising AFs to reduce the gasoline and diesel consumption and air pollution from the transportation sector. Despite the huge reduction in air pollution by these AFs, they have not been used widely by users around the world mostly due to their high prices and availability concern compared to gasoline and diesel vehicles. If governments explain correctly the impacts (short and long-term) of air pollution on human health and the environment to the users, as well as providing some incentives for the users of AFVs, they will select the AFVs even the prices are higher than gasoline and diesel vehicles. For instance, currently, people around the world are trying to find protection items (e.g., face mask, hand wash liquid, etc.) regardless of their huge prices during the COVID-19 outbreak period. Because they understood that their lives might be in danger; therefore, they are purchasing protection items that are currently more expensive than before the COVID-19 outbreak period. Also, since the demand for the protection items is rising; thus, the manufacturers are increasing their potential to produce more items then before. This means that the demand is the most important factor followed by other factors like price. In another example, those people who have been facing air pollution issues and have environmental concerns, like Indian people, agree to buy AFVs even their price will be higher than conventional gasoline and diesel vehicles as mentioned in Table 2 for Refs. [100, 101, 102]. Because they found that their country suffers from air pollution (see the PM_2.5_ levels per country/region as one of the main sources of air pollution in Figure 7) and the changes in road vehicles standards (such as using alternative and clean fuels, after-treatment systems, frequently vehicle maintenance, etc.) will help in the reduction of air pollution as shown in Figure 8. According to the literature (see Figure 8), among the existing technology and policy solutions for cleaning the air in Asia (as an area having many countries with a high level of PM_2.5_ as listed in Table 2), more attention on emissions regulations and using alternative fuels for vehicles and industries can be one of the most effective methods in the reduction of PM_2.5_ for the present and even future (2030). The very low impact of electric cars on the reduction of PM_2.5_ in Figure 8 is due to their low contributions in the Asian transportation fleets which need to be increased (as an objective of this review). It will be important to inform people to know that the motor vehicles and manufacturing facilities using fossil fuels are the main sources of ambient air pollution in their countries [20, 116, 117].

Therefore, if each person finds out that the world is suffering from air pollution and understands correctly his/her responsibilities for human health and the environment, he/she will participate in the usage of AFs (regardless of their price) to reduce air pollution. And the manufacturers of AFs can drop the final product price (as well as an increase in product quality) due to more production as a consequence of the rise in the demand.

In another example, people around the world spend their money on some items that may not be very necessary for their life. Pet rock, motorized ice cream cone, among others, can be exampled. The pet rock is only a small simple raw egg-shaped stone taken from rivers and beaches which is delivered to the consumers with a cardboard box (with breathing holes to show to the consumers that this stone can be counted as a real pet), straw, and a manual (some notes to teach users how to care and train the stone). The pet rock's founder invited people to have a pet that had no need for food and water, and it would not be ill or die [120]. For a short period (about six months), more than 1 million of pet rocks were sold in 1975 (the first year of production) for USD 3.95 each [121]. Similar products even with accessories (like pet leash for stone, etc.) are still available on the market. Motorized ice cream cone (US Patent in 1999 [122]), as another example, is a hand-held device that spins the ice cream cone; thus, the person who wants to eat an ice cream cone, only fixes his/her tongue on the ice cream cone. This product is also still available on the market for few dollars. We don't want to mention in this review that these products are useful/useless or cheap/expensive for human life, we just exampled them to emphasize that people spend money on some products which may not be very necessary for their lives and money is not a big issue for the selecting some products. And the inventors of these products introduced them as necessary needs (may be true or not) for the societies, but of course with a good advertisement. We know that motor vehicle is more expensive compared with these products exampled above, however, we have mentioned these examples to emphasize that advertising plays a key role for selling a product followed by other factors, thus, governments and policymakers may increase the AFs' advertisement potential for achieving more adoption of AFs. Because, according to the findings from the systematic literature reviewing 43 papers before 2017 as listed in Ref. [123] and other studies published after 2017 [71, 94, 123, 124, 125, 126, 127] from different countries, most of the people who receive good information, news, and advertisement from their friends, parents and family members, relatives, neighbors, colleagues, media, etc. about AFVs have more intention to use and purchase them rather than conventional vehicles.

As the last example, there were almost no significant demands (except few demands from rich people) for buying vehicles during their first years of invention and manufacturing period, due to some concerns by people, including their cost, maintenance, safety, and fuel availability compared to those of coach (carriage) with horses as the main transportation system. However, after few years, people found that the vehicles can be useful for their lives (there were no big concerns about air pollution and GHG emissions in those periods compared with nowadays), although those vehicles were very costly, and the fuel availability was not good as that for the coach. Thus, people started to purchase vehicles. A similar case is happening in recent years to shift conventional gasoline and diesel vehicles to clean vehicles.

**Figure 9:**
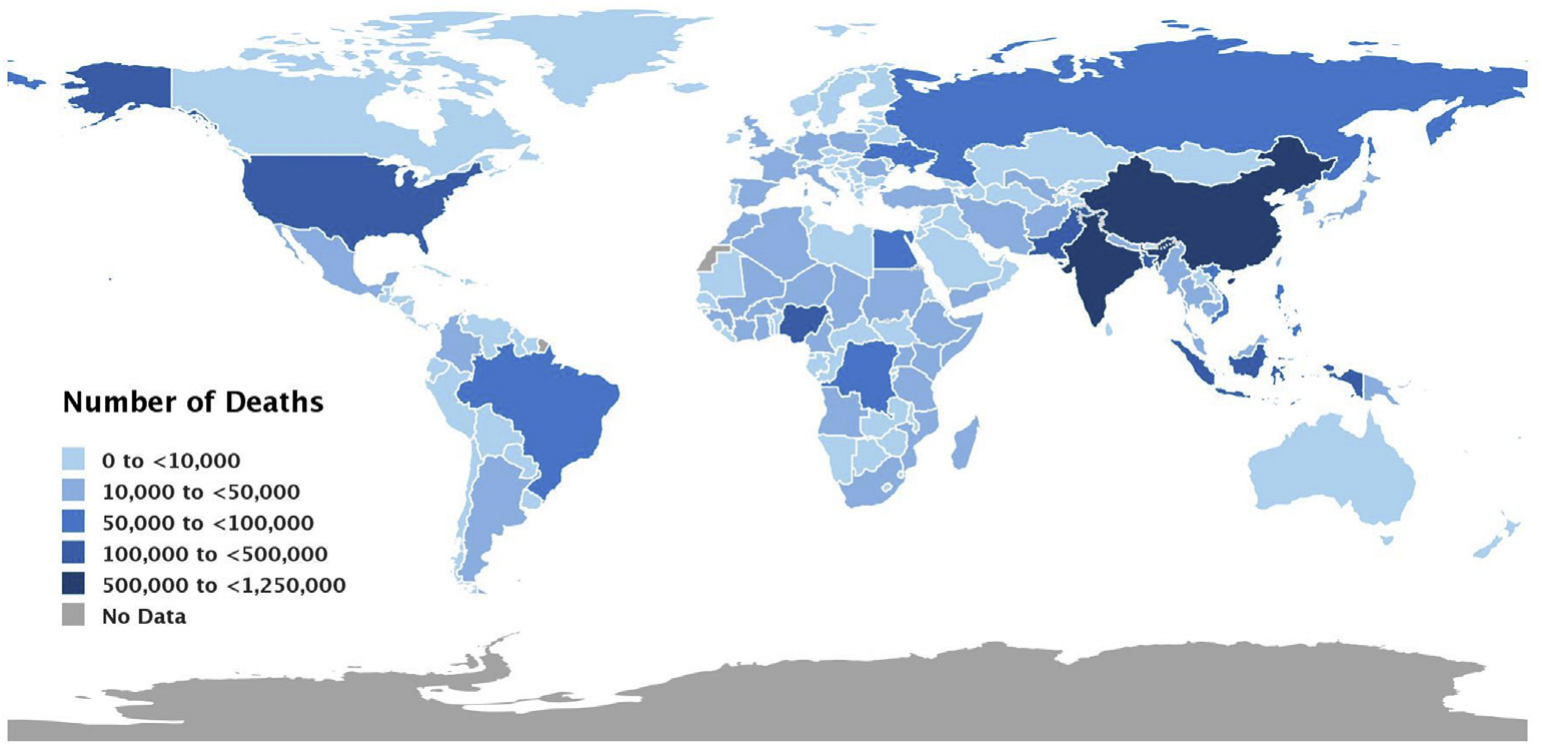
Number of deaths attributable to air pollution around the world in 2017 [116]. Note: according to the publisher's policy for the State of Global Air report, as an open-access source, regardless of requirement for a proper citation, no permission is required to reprint the figure.

**Figure 10:**
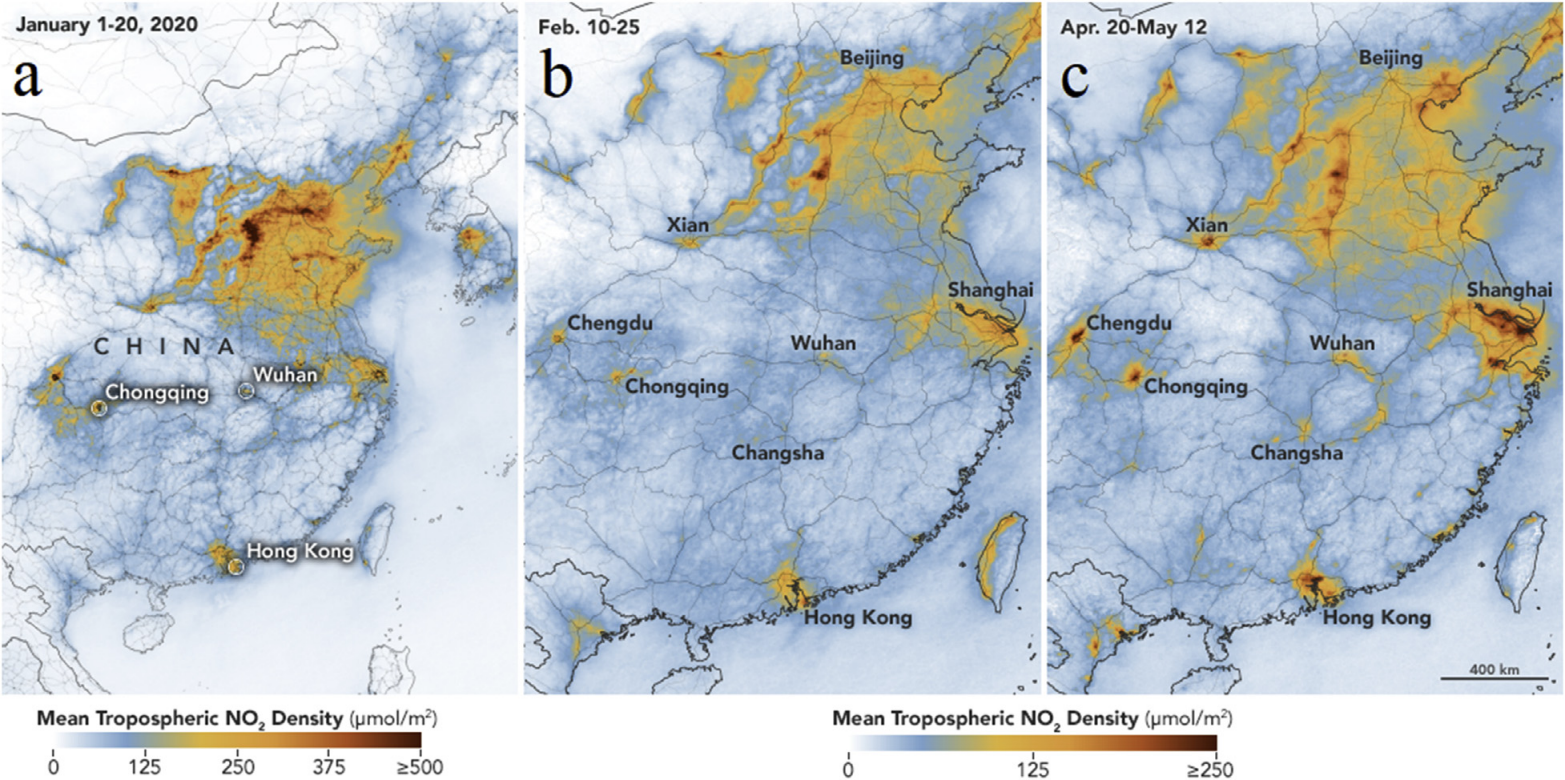
Effect of lockdown for some industries and transportation during the COVID-19 outbreak on NO_2_ concentration in major cities of China, as: a) 1–20 January 2020 (before the lockdown period), b) 10–25 February 2020 (during the lockdown period) and c) 20 April - 12 May 2020 (after the lockdown period), maps were taken by NASA [133, 134].

It will be very useful to inform people about the effect of air pollution and GHG emissions on human health and the environment. According to the data from World Health Organization (WHO), air pollution (ambient plus household air pollution) causes stroke, heart disease, chronic obstructive pulmonary disease, lung cancer, and acute respiratory infections, resulting in about 7 million deaths per year in the world in recent years, because 9 out of 10 people breathe air containing high levels of pollutants. In detail, ambient air pollution caused about 4.2 million deaths, and household air pollution from cooking with polluting fuels (fossil fuels especially coal, wood, biomass, etc.) and technologies caused about 3.8 million deaths in 2016 (note that 2016 was the latest and updated year for WHO's reports in 2020). Also, currently, air pollution is shortening human's life on average by 20 months and it is counting as one of the leading risk factors for global mortality (about 14% of total global deaths in 2016 [128] and known as the fifth leading risk factor in 2016 and 2017 [116, 128], see Figure 9 for the number of deaths per each country) which is more dangerous than road traffic mortality (about five times in 2016) [116, 128]. On the other hand, the WHO data shows that climate change, as a consequence of rising in GHG emissions, has an effect on global human mortality and it is expected that it will cause about 250,000 additional deaths per year, from malnutrition, malaria, diarrhoea, and heat stress, between 2030 and 2050, and it will cause direct damage costs of about USD 2–4 billion/year by 2030 to health (excluding the costs in health-determining sectors such as agriculture, water, and sanitation) [129]. It can be useful to know that the motor vehicles and manufacturing facilities using fossil fuels are the main sources of ambient air pollution as well as GHG emissions [20, 116, 117]. It is reported that the reduction in industrial and road traffic emissions such as CO_2_, nitrogen oxides (NO_X_) and related ozone formation, and particulate matters can improve the air quality. For example, the air quality levels in the world's major cities improved dramatically (global air traffic dropped by 60%) [130], or 30% less in nitrogen dioxide (NO_2_), see Figure 10 (a and b), levels and 25% less in CO_2_ in China (equals to about 100 Megatons of CO_2_) [131,132] in March and April 2020 over the same period in 2019 due to reduction in industrial and road traffic emissions from fossil fuels, because of a short lockdown period (one to two months) for some industries and reducing the travels and traffics on the roads during the COVID-19 outbreak. However, the NO_2_ concentration is increasing after resuming the industries and transportation duties in China after the lockdown period, as shown in Figure 10 (c), while scientists expected this rebound. Thus, reduction in using fossil fuels due to using AFVs can have a significant effect on the reduction of both air pollution and GHG emissions in a long-term. Thus, if we educate people that their gasoline or diesel vehicles can have a contribution on shortening their or their families or other individuals' lives (by 20 months on average), or causing death for humans, therefore, they will pay more attention to use AFVs.

Thus, we can understand that governments and researchers can explain comprehensively to people that the use of AFVs is a necessary need (which is 100% true) for our world for the reductions of air pollution and GHG emissions, even there is a high vehicle purchase cost (about 20–300% for some AFVs compared to gasoline and diesel vehicles in recent years [26], while most of these AFVs can return money back in short/long-term use due to their lower fuel price and maintenance cost or having subsidies from governments [26]). According to Table 2, some people around the world have willing to pay more money for AFVs than conventional vehicles to protect the environment and human health. Although there are some issues rather than the only price on AFs, including safety and fuel availability, governments can encourage people via the following recommendations to use those AFVs which have tackled the technical, safety, and availability issues (through encouraging AFVs manufacturers by governments and increasing in AFVs’ demand by the users), while their high price is the only left issue:

• Increasing people's knowledge about the latest performance of AFVs through social media; many people, drivers, and car owners in the world (Table 2) still do not select AFVs, because they have old information (or no/less/wrong information) about the AFVs which is not completely correct for the present time, such as their purchase price, fuel economy, fuel price, driving range, refueling/recharging stations, their effect on the environment, etc.; since society (including social media, family, friend, neighbor, etc.) has a significant effect on global diffusion of AFVs [71, 94, 123, 124, 125, 126, 127], therefore, it is recommended that governments can use social media for this purpose, especially advertisements during family or female programs on TV, because females have more intention (but less knowledge) than males to use AFVs, particularly EVs (Table 2);
• Increasing people's knowledge about the period (e.g., years) required for payback of higher AFVs prices compared to gasoline and diesel vehicles, from fuel savings, maintenance, repairing, or other ownership costs of AFVs; because several drivers are willing to buy AFVs if their payback period is one or two years [135];
• Increasing people's knowledge about the impact of air pollution on human health and the environment through social media (TV, internet, newspaper, etc.), unfortunately almost all the current advertisements are about food (like potato chips, etc.), drink or house materials in social media and there is almost no advertisement in social media about air pollution in the world and human's effect on it;
• Increasing people's knowledge about their responsibilities for the present and the future on human health and the environment through social media;
• Allocating some information about the impact of air pollution on human health and the environment, and the positive influence of AFs on the reduction of air pollution in educational lessons and books for students; because young and high-educated people have more willing to use AFs compared to older and less-educated people (Table 2);
• Informing people, in detail, that some AFs might have higher purchase cost than gasoline and diesel vehicles at this moment, while some of them can return money back in long-term use, due to their lower fuel price and maintenance cost or having subsidies from the government; for example, currently the US and Chinese governments give some subsidies which dropped the purchase price of several models of EVs to sometimes more than half of their original prices [136]; so this type of subsidies can be used for other countries as well;
• Using AFVs in movies or TV series;
• Adding AFVs in computer games [137];
• Reducing or waiving tax, insurance, and toll costs for the vehicles using AFs; for example, Norway, as one of the most successful deployments of EVs in terms of market share in the world, has planned for tax exemption for vehicle and low annual road tax, free municipal parking, toll-free roads or ferries, and free public charging stations for EVs, to encourage people to use EVs [138, 139];
• Using AFVs in the car sharing fleets can diffuse them in the world. Because, on the one hand, 50.4 million users (332 thousand vehicles) globally used vehicles from the car sharing fleets in 2018 and it will reach about 227 million users (1.2 million vehicles) [140] which is a great potential for illustrating the advantages of AFVs over gasoline and diesel vehicles to people. On the other hand, those people who used vehicles from car sharing fleets can recommend other people (or themselves) to purchase those vehicles in the future, for example, in a study from the USA in 2020 [141], more than half of PHEVs' or EVs' users (3662 participants) who used PHEVs or EVs from the car sharing fleets, would recommend others to purchase them, while only about 3% of participants did not agree to recommend them to others, and the rest participants had a neutral opinion;
• Registration fee reduction for the vehicles using AFs;
• Free or ease access to getting vehicle license plate for the countries in needed; for example, in some megacities in China, e.g., Beijing, the vehicle license plate value is very costly and can reach more than USD 19,000 (price in 2018–2019) and it needs time to obtain (from several days to several years, due to license plate lottery) for traditional vehicles, so the Chinese government has waived these to difficulties for EVs in Beijing or other megacities to encourage people to use EVs, so EV owners in several megacities in China can save at least USD 10,000 (on average) [136];
• No limitation or more freedom for entering zero emitter vehicles (e.g., BEVs) on the road; for example, the vehicles which have odd (or even) number in their vehicle license plate (or using other methods) are not allowed to enter on the road, in some days of the week, to reduce air pollution in some cities (e.g., Beijing in China);
• Allocating specific parking area for the vehicles using AFs [96], especially in those countries suffering limited parking areas;
• Reducing parking fee for the vehicles using AFs, especially in those countries suffering high parking fee; for example, government car parking fee for private cars and vans in Hong Kong (as one of a dense places in the world) is reached up to about USD 23 per hour, during day period, in some places in 2021 [142] and it will be more expensive in the future; while currently, USD 23 is equal to about 9 or 11 L of gasoline or diesel fuel, respectively in Hong Kong (having the most expensive gasoline and diesel in the world), or about 19 or 22 L of gasoline or diesel fuel, respectively for around the world (based on the average price on 28 June 2021) [22].
• Allocating some prizes randomly for some buyers of the vehicles using AFs;
• Installment plan, discount and rebate, and profit for buyers of the vehicles using AFs [71];
• Allocating some grants, vouchers, low-cost loans, or tax credits for users of the vehicles using AFs [26];
• Existing subsidies on gasoline and diesel fuels in several countries is a barrier to adopt AFs [24];
• Increase in alternative fuel and charging stations for easy accessibility [143, 144];
• Offering free electricity or lower price charging stations for EVs [49, 145];
• Offering cash incentives to people to purchase AFVs, for example, currently, the government of Germany under the “Environment Bonus” plan, is starting to give incentives (up to about USD 7,000 per vehicle) for hybrid vehicles and EVs [146], or USD 2,500 to USD 7,500 federal tax credit for plug-in hybrids and all-EVs in the USA [29]; so this type of incentives can be used for other countries as well;
• Free or reduction in cost for converting traditional vehicles to AFVs using gas or biofuels;
• Providing vehicle-to-grid (V2G) system for EVs;
• Providing stable and long-term policies regulatory framework for AFVs (i.e., 10–15 years and more) [13];
• Supervising after-sales service quality and providing various purchase channels [147];
• Shortening the time required (sometimes 3–4 month waiting periods) for receiving some AFVs after ordering at the car dealerships [92];
• Arranging vehicle showcasing events and offering AFVs at the car dealerships for people to free test-drive AFVs [92];
• Increasing car salespeople's knowledge about the real, updated, and beneficial specifications of AFVs over traditional vehicles [87, 92].

The recommendations above may be selected and implemented by governments according to the developing levels of their countries and the people's lifestyles. For instance, the increase of people's knowledge about the AFs and air pollution may be more useful and effective than cost benefits in developed countries; while recommendations related to the cost benefits may be more useful and effective than people's knowledge in less developed countries. It is noticeable to mention that people around the world can be encouraged to use AFs when governments themselves are firstly encouraged. This issue can be mostly seen in the less developed or underdeveloped countries. Because some of the governments in these countries do not accept or do not want to accept (may be due to financial issues) that air pollution is a real threat for the world which can be treated only with cooperation with other countries simultaneously. Therefore, they have no attempt to use the AFs.

In addition, governments should focus on both demand by consumers and supply sides, simultaneously, to motivate both automaker investment in AFVs and consumer purchase intention [136]. Thus, governments can encourage the producers of AFs to produce safer and cheaper and more available AFs to support the consumers’ demand via the following recommendations:

• Reducing tax for the producers of AFs;
• Reducing advertisement cost for the producers of AFs in social media;
• Using waste and non-edible feedstocks like waste cooking oil, algae, biomass, etc. for biofuel production; because several people prefer to use non-edible sources which are more eco-friendly materials and have no influence on food price and availability compared to edible feedstocks;
• Allocating some specific research funds for AFVs; in recent years, most of the funds are provided by manufacturers themselves, so governments can help and support them by providing financial and non-financial materials
• Long-term sales contracts
• Guarantee to purchase (or financial support) those remaining products of manufacturers which are not purchased by people;
• Most of the current AFVs, particularly EVs, have a simpler design and shape with fewer features than conventional vehicles, resulting in less attention of several people to purchase AFVs; so it is recommended to solve these deficiencies of AFVs.

Although governments should spend some money for the recommendations above; but on the other hand, the reduction in the requirements for air cleaning facilities, vehicle emissions catalysts, filters, etc. will offset those expenses. For example, emission catalytic converters (turning the harmful emissions from vehicle exhausts into harmless components) used for ICEs cause an increase in vehicle cost by a few hundred dollars to a few thousand dollars (sometimes to about USD 3,000) depending on the type and age of vehicle, country, etc. Also, they have taken a significant budget of governments. The consumption and investment budget for the global automotive catalytic converter market is projected to be several billion dollars, e.g., up to about USD 300 billion, by 2025 with a compound annual growth rate of mostly about 8% [148, 149, 150, 151]. Note that those budgets are based on the information about a few types of catalytic converters, including two-way catalytic converter, three-way catalytic converter, four-way catalytic converter, diesel oxidation catalyst, and selective catalytic reduction, and information mostly from the USA, Canada, the UK, Germany, France, Italy, Spain, China, India, Japan, South Korea, Brazil, Argentina, Mexico, and Middle-East, and a few countries from Africa, that use the emission catalytic converters more than other countries. Thus, the information for more types of catalytic converters coupled with more countries will increase those budgets accordingly.

Also, with the application of those AFVs emitting zero emissions (e.g., battery electric vehicles) in the transportation sector, there will be no needs for spending those budgets for installation and maintenance of emission catalytic converters, and also they can combat the disadvantages of emission catalytic converters (e.g., effects of catalytic converters on vehicle performance, fuel consumption and formation of CO_2_ as a GHG by CO conversion in catalytic converters [152, 153, 154]). While the global EV charging infrastructure market is much less than that of the global automotive catalytic converter market. For the leading countries to use EVs, including the USA, Canada, the UK, Netherlands, France, Norway, Germany, China, Japan, and South Korea, the vehicle charging infrastructure market for commercial and residential applications with fast and slow charging types, will be estimated to be less than USD 20 billion by 2025 with a compound annual growth rate of about 32% [155]. Thus, shifting the investment budget from catalytic converters to EV charging infrastructure can be a good option to increase the number of electric charging stations for EVs to encourage people to use EVs and rise in EVs’ demand volume.

## 7 Conclusions

Currently, we, including governments, researchers, and producers of AFVs around the world are trying to find some solutions for replacing gasoline and diesel vehicles (for light- medium-, and heavy-duty applications) with AFVs to reduce air pollution and GHG emissions followed by dependency in the fossil fuels consumption. However, almost all the solutions are related to the technical and complicated aspects' side, like improvement in technologies, but there is rare attention to the human behavior's side (like knowledge, education, promotion, environmental issues, etc.) which is a critical point for the increase in the number of AFVs in the global transportation sector. Waiting for the reduction in the price of AFVs and the rise in their availability as a consequence of more technology improvement might be a primary solution, but it cannot guarantee that the consumers will buy them in the future. Because the technologies on conventional gasoline and diesel vehicles will also be improved in the future, resulting in changes in their fuel economy, performance, and emissions, and also their more global availability (especially in less developed countries which have a significant population in the world). Thus, we should look for a permanent solution along with its simplicity/applicability for the whole world. The solution can be simple as the famous solution for kicking out ants from houses. That is only a sentence as “remove the food in the house which ants are fed with that”, therefore, the ants will permanently leave the house by themselves without the needs for any chemical material (as a temporary solution). Yes, the permanent solution could be that simple for the transportation sector as well. The solution is that we should encourage (not force) and educate people and car salespeople to use (sell) AFVs rather than only waiting for more improvement in technologies. If they could be well educated by us with receiving some help from governments (like financial and non-financial incentives), they will embrace and purchase the AFVs by themselves in the future for sure. Thus, to sum up, it is recommended that governments could pay more attention to use their financial and power policies to encourage and educate people to use AFVs, rather than only supporting and focusing on industries and research sides to develop the technologies to reduce the vehicle and fuel prices, because many people will not choose AFVs even when their price and performance be the same as those of gasoline or diesel vehicles due to their little knowledge about AFVs. Otherwise, without making people ready to use AFVs, the achievement of a significant increase in the number of AFVs in the global transportation sector will be postponed year by year or even decade by decade.

## Declarations

### Author contribution statement

All authors listed have significantly contributed to the development and the writing of this article.

### Funding statement

This work was supported by the 10.13039/501100004733University of Macau, The Hong Kong Polytechnic University, and The Hong Kong University of Science and Technology.

### Data availability statement

Data included in article/supp. material/referenced in article.

### Declaration of interests statement

The authors declare no conflict of interest.

### Additional information

No additional information is available for this paper.

## Footnotes

1 Evolving Transition Scenario assumes that the evolution in government policies, technologies, and societal preferences will be in a manner and speed similar to the recent past.

2 Reference Case Scenario refers to the current trends and relationships among supply, demand, and prices in the future with including some anticipated changes over time like changes in regional economic and demographic trends, infrastructure, and incremental cost and performance improvements in known technologies.

3 Sustainable Development Scenario is a roadmap for meeting international climate and energy targets to achieve net-zero emissions in the future (like 2050).

4 Reference Technology Scenario refers to today's commitments by countries to limit emissions and improve energy efficiency, including the Nationally Determined Contributions pledged under the Paris Agreement (keeping the rise in the global average temperature to well below 2 °C above pre-industrial levels).

5 2 °C Scenario lays out an energy system pathway and a CO_2_ emissions trajectory consistent with at least 50% probability to limit the expected average global temperature increase to 2 °C by 2100.
